# Colloidal AgBiSe_2_ and AgBi(S_1–*x*_Se_*x*_)_2_ Nanocrystals with Composition-Tunable Properties Based on Bis(acyl) Selenide Precursors

**DOI:** 10.1021/acsomega.6c04337

**Published:** 2026-07-21

**Authors:** Julia Prodan, Anatol Prudnikau, Falk Röder, Xuan Qi, Fabian Paulus

**Affiliations:** † 28394 Leibniz Institute for Solid State and Materials Research (IFW) Dresden, Helmholtzstraße 20, 01069 Dresden, Germany; ‡ Leibniz Institute of Polymer Research (IPF) Dresden, Hohe Straße 6, 01069 Dresden, Germany

## Abstract

Heavy-metal-free metal selenide nanocrystals have attracted great interest as low-cost, environmentally friendly, and solution-processable materials for future optoelectronics and thermoelectrics. Despite the widespread prospects of these materials, their preparation and processing remain a challenge, as the fabrication is laborious and may require harsh environments and toxic chemicals, and, more importantly, most widely used selenium precursors for the synthesis of colloidal NCs are not only toxic and expensive but also highly unstable, which makes them extremely difficult to handle and leads to unreproducible results. In this work, we present a facile synthetic approach for obtaining colloidally stable ternary silver bismuth selenide (AgBiSe_2_) and quaternary silver bismuth sulfide selenide (AgBi­(S_1–*x*_Se_*x*_)_2_) nanocrystals by utilizing bis­(acyl) selenides as an alternative precursor in the hot-injection synthesis method. The employed bis­(acyl) selenide precursors are stable under ambient conditions, easily processable, and allow great composition control on the anion side and, consequently, band gap fine-tuning in the case of AgBi­(S_1–*x*_Se_*x*_)_2_ nanocrystals, making them highly attractive for solution-processed optoelectronic devices.

## Graphical abstract

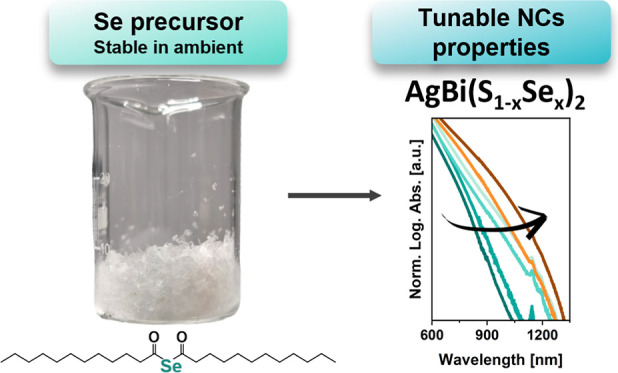

## Introduction

Colloidal semiconductor nanocrystals (NCs) have attracted significant attention in the past several decades due to their tunable band gap and unique optical properties, which makes them promising contenders for potential applications in optoelectronic devices, such as photovoltaic and photodetector devices.^1–3^ In particular, silver bismuth disulfide (AgBiS_2_) nanocrystals have been notably studied and demonstrate high absorption coefficients, phase stability, and a direct bandgap (1–1.3 eV), which makes them ideally suited for applications in optoelectronics, particularly solar cells.^4–9^ Furthermore, the constituent elements of this material are relatively earth-abundant, cost-efficient and considered environmentally friendly and low-toxic, especially when compared to commonly used and widely studied heavy-metal-containing materials such as PbS, CdTe, and lead-based perovskite materials.^10–13^ Additionally, AgBiS_2_ NCs do not require high-temperature synthesis and thus could potentially provide short energy-payback times, unlike commercially available silicon solar cells that rely on extremely high processing temperatures and possess energy payback times in the order of 12–18 months.^14–16^ Nevertheless, AgBiS_2_ NCs exhibit a spectral response only in the visible and near-infrared (NIR) until ca. 1100 nm. The rising need for NIR optoelectronics, particularly photodetectors, in medicinal and sensor applications in turn increases the need for new materials with the extended spectral response deeper into the infrared.^17^ Additionally, the commonly studied materials for applications in NIR devices also often rely on highly toxic heavy metals, such as Hg and Cd,^18,19^ or moderately toxic rare-earth elements, as seen in III–V group compounds, namely InGaAs, which is used in commercially available photodetectors and photodiodes.^20–22^ Silver bismuth diselenide (AgBiSe_2_) NCs are a viable alternative material system, combining the advantages of AgBiS_2_ NC as a more environmentally friendly, cost- and energy-efficient, and earth-abundant material composition with an optical response further into the NIR. AgBiSe_2_ NCs also exhibit a rock salt crystal structure similar to AgBiS_2_ NCs, allow for low-temperature processing, and provide desirable electronic and optical properties, such as high absorption coefficients, as well as a direct bandgap in the range of *E*_g_ = 0.7–1.0 eV,^23,24^ in comparison to bulk AgBiSe_2_ with a bandgap of *E*_g_ = 0.6 eV.^25^ By employing mixed compositions in the form of AgBi­(S_1–*x*_Se_*x*_)_2_, fine-tuning of the band gap and the absorption edge can be realized while maintaining the small NC size.^26^ However, the main issue for synthesizing selenium-based NCs is the unavailability of an air-stable, cost-efficient, or adequately reactive selenium precursor, which often leads to the need for complex or extreme reaction conditions and setups in order to obtain these NCs. In most cases, elemental selenium powder is used for the synthesis of selenium-based NCs, usually in alkyl phosphine complexes such as with TOP or TBP.^27–29^ Alternatively, in situ reduction of selenium oxide is achieved in the presence of thiols and amines, and results in colloidally stable polyselenides. Most often, selenium-containing precursor solutions are obtained by using long-chain organic fatty acids and derivatives, such as oleic acid (OA), oleyl amine (OAm), or octadecene (ODE).^30,31^ However, these precursor solutions are oftentimes susceptible to air and prone to quick oxidation and degradation. Therefore, they have to be prepared in situ or typically shortly before nanocrystal synthesis and must be handled under inert conditions, further complicating the synthesis. In contrast, the group of Norris utilized several organo-selenium precursors for the synthesis of CdSe nanoplatelets, namely bis­(stearoyl) selenide, bis­(benzoyl) selenide, and bis­(4-chlorobenzoyl) selenide.^32^ These substances are air-stable solids, which dramatically simplify handling and up-scaling. Additionally, we recently showed that similar bis­(acyl) sulfide precursors can be utilized for synthesizing high-quality nanocrystals, such as PbS, AgBiS_2_, and Ag_2_S NCs while providing narrower size distributions compared to other chalcogenide precursors.^33,34^ However, the use of the respective selenides as solid, air-stable, and easy-to-handle selenium precursors remained unexplored for ternary AgBiSe_2_ NC and, particularly, the mixed, quaternary silver bismuth sulfide selenide AgBi­(S_1–*x*_Se_*x*_)_2_ NCs.

In this work, we report on the synthesis of NIR-active AgBiSe_2_ NCs by utilizing three different bis­(acyl) selenides, namely, bis­(benzoyl) selenide (Bz_2_Se), bis­(stearoyl) selenide (St_2_Se), and, so far, not reported bis­(lauroyl) selenide (La_2_Se), as solid, air-stable but reactive and easily processable precursors. We study the precursor-dependent properties of the resulting nanocrystals, which exhibit sizes ranging from 3.8 to 5.5 nm. By mixing the bis­(acyl) selenide precursors with sulfur-based precursors in the nanocrystal synthesis, quaternary AgBi­(S_1–*x*_Se_*x*_)_2_ NCs with constant average sizes around 3.8 nm but adjustable anion composition and, therefore, fine-tunable optical properties can be realized. Such bandgap-tunable environmentally friendly nanocrystals are promising semiconductors for future solution-processed NIR optoelectronics.

## Results and Discussion

To circumvent the use of highly reactive, air-sensitive selenides or the in situ preparation of selenide-containing reaction media, bis­(acyl) selenides were used as a selenium source in the classical hot-injection synthesis method. In particular, bis­(stearoyl) selenide (St_2_Se), bis­(lauroyl) selenide (La_2_Se), and bis­(benzoyl) selenide (Bz_2_Se) were used as air-stable selenide precursors (Figure 1a) for the formation of silver bismuth diselenide (AgBiSe_2_) nanocrystals. These selenium precursors can be obtained by the reaction of the corresponding product of selenium reduction (LiAlHSeH) by lithium aluminum hydride (LiAlH_4_) in tetrahydrofuran (THF) under cooling.^35^ Purification of these selenium compounds can be realized by simple recrystallization, for example, in diethyl ether. While the synthesis of St_2_Se and Bz_2_Se has been previously reported elsewhere,^32^ we realized the synthesis of La_2_Se as a selenium precursor with a medium chain length. The corresponding ^1^H-NMR, ^13^C-NMR, and FTIR spectra for La_2_Se can be found in Figures S1 and S2. All resulting bis­(acyl) selenide precursors are air-stable, colorless powders, which are well-soluble in most organic solvents. The main advantage of these synthesized bis­(acyl) selenides is that they are not prone to quick oxidation like many other commercial selenium precursors or in situ prepared (poly)­selenide-containing solutions. They also can be stored “ready for use” for several weeks in closed vials in the normal laboratory environment, greatly simplifying the synthesis of selenium-containing NCs. In order to demonstrate the long-term stability of the selenium precursor, particularly La_2_Se, ^13^C-NMR was conducted on a fresh synthesis and, afterward, on the same synthesis after 2 months of storage in ambient conditions. The stacked ^13^C-NMR spectra, which are given in Figure S3, clearly depict no detectable changes, indicating excellent long-term storage stability even in ambient conditions. For the synthesis, the selenium precursors are dissolved in the corresponding organic solvent (toluene) and injected into an outgassed solution of bismuth and silver acetates in a mixture of OA and ODE at 100 °C. The reaction occurs instantaneously, and the solution turns dark brown, highlighting the high reactivity of the bis­(acyl) selenide precursors. The formation of AgBiSe_2_ NCs requires the use of 1-dodecanethiol (1-DDT), which stabilizes the NCs and limits their growth. Synthesis without 1-DDT results in large AgBiSe_2_ NCs that tend to aggregate and precipitate within a few seconds to minutes (Figure S4). Following transmission electron microscopy (TEM) analysis, the synthesized AgBiSe_2_ NCs exhibit an average size of 2.6, 3.8, and 5.2 nm for St_2_Se, La_2_Se, and Bz_2_Se, respectively (Figure 1b-d). The average NC size increases with reduced chain length of the acyl moiety, suggesting that the precursors’ reactivity and the NCs’ steric passivation during the growth phase might be affected by the molecular structure of the selenide precursor. We speculate that the corresponding organic acids, which can form as a potential main product of the precursors’ decomposition, could serve as ligands similar to oleic acid and, in turn, influence the nanocrystal sizes. Our AgBiSe_2_ NC sizes are noticeably smaller compared to AgBiSe_2_ NCs obtained by the more established selenium sources, highlighting an additional benefit of these selenide precursors.^26^ The standard deviations of the size distributions are below 1 nm in all cases, and the corresponding histograms can be found in Figure S5. The La_2_Se-based AgBiSe_2_ NCs provide the narrowest size distribution, beneficial for application in optoelectronics. X-ray diffraction (XRD) measurements were conducted to probe the structural properties of the synthesized NCs (Figure 1e). The drop-casted NC dispersions all exhibit a diffraction pattern in agreement with the expected reflections for AgBiSe_2_ ^36^ and are significantly broadened due to the small nanocrystal size. The XRD pattern of NCs synthesized with St_2_Se also exhibits undesirable reflections, with a most prominent one around 36°, which could potentially be attributed to the formation of phase-separated Ag_2_Se (ISCD, code 177917). However, such impurities are not visible in the case of using La_2_Se and Bz_2_Se. The ultraviolet visible absorption (UV/vis) spectroscopy measurements of the NCs in solution exhibit featureless absorption curves (Figure 1f), which are typical for silver bismuth chalcogenides.^26,34^ Only in the case of St_2_Se some shoulders can be observed, which most likely originate from the Ag_2_Se impurities as discussed above. The absorption extends substantially further into the NIR compared to AgBiS_2_ nanocrystals, resulting in an estimated bandgap of around 0.92–0.95 eV for the AgBiSe_2_ nanocrystals.

**Figure 1:**
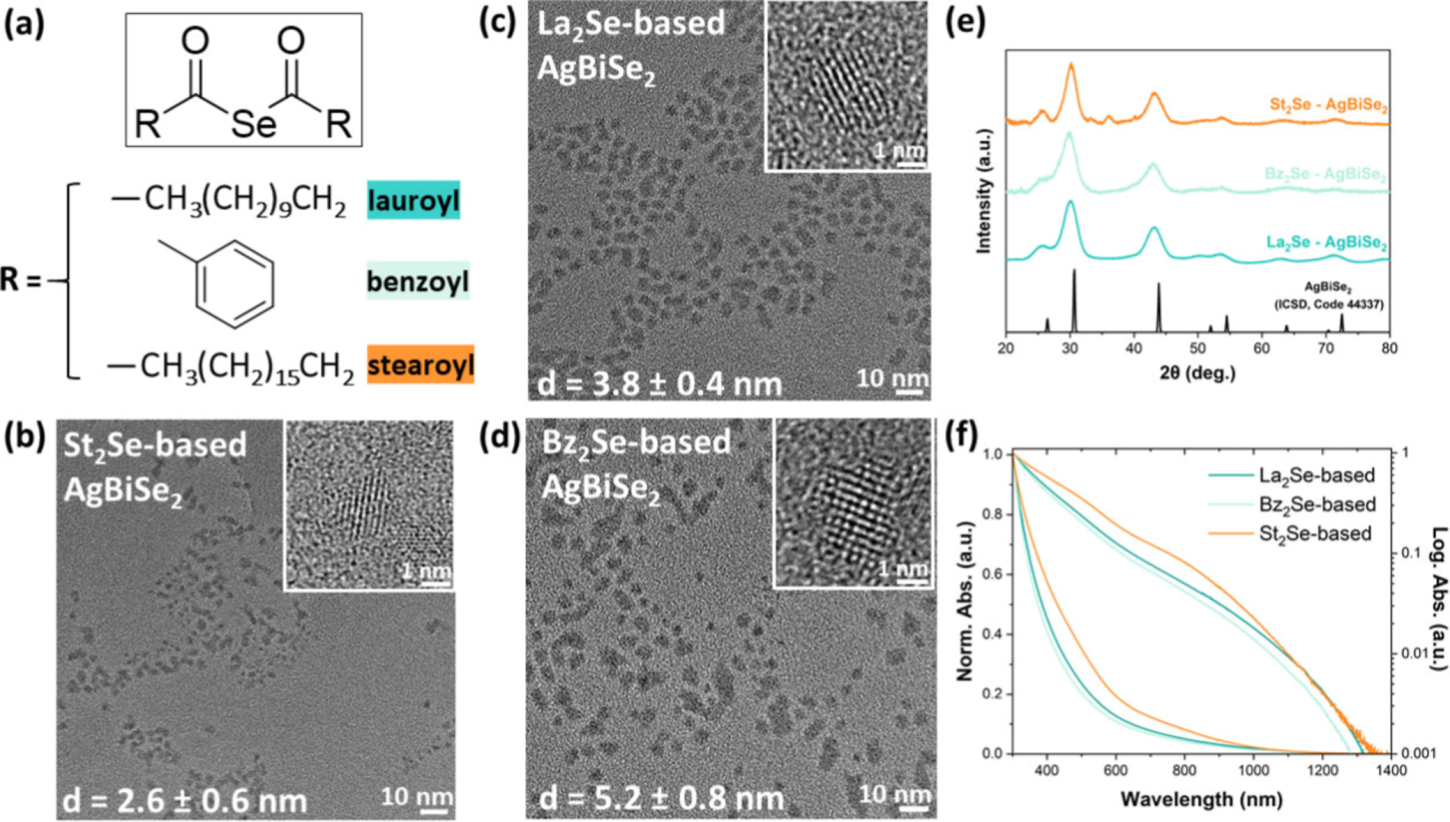
Characterization of AgBiSe_2_ NCs synthesized with different bis­(acyl) selenide precursors. (a) Chemical formulas of bis­(acyl) selenide precursors used for the NC synthesis. TEM overview images and high-resolution images (inset) of AgBiSe_2_ NCs synthesized with (b) St_2_Se, (c) La_2_Se and (d) Bz_2_Se. (e) XRD patterns of synthesized AgBiSe_2_ NCs with the different bis­(acyl) selenide precursors. (f) Normalized optical absorbance spectra of the synthesized AgBiSe_2_ NCs for the three different selenium precursors in solution, plotted in both linear and logarithmic scale.

We attempted to tune the size of AgBiSe_2_ NCs with different methods, utilizing the La_2_Se precursor. Increasing the reaction time up to 4 h does not show any significant size change, as confirmed by TEM, UV/vis, and XRD analysis (Figure S6). By attempting to increase the reaction temperature, we found that a reaction conducted at 150 °C, while possible, shows inconsistent particles incomparable to synthesis conducted at 100 °C and demonstrates quick aggregation (Figure S7). Other attempts to synthesize at higher temperatures (180 °C, 200 °C) showed immediate aggregation in the flask upon hot injection. Another parameter to modify the NC size was the time of 1-DDT injection; however, with longer injection time delays, UV/vis spectra no longer exclusively correspond to AgBiSe_2_ NCs, especially with the appearance of a prominent peak at ∼360 nm and between 1100 and 1200 nm. Additionally, XRD patterns confirm the formation of other phases as the main product (Figures S8 and S9). Lastly, also reducing the concentration of 1-DDT to the growth of larger NCs during the hot injection led to immediate aggregation of the resulting NCs. Evidently, the strong surface passivation, which is provided by 1-DDT, drastically limits the potential size tuneability of the NCs, which is confirmed by the lack of particle growth independent of growth time and temperature. Furthermore, the necessity of immediate injection of an appropriate amount of 1-DDT into the system is apparent, as the results show that an injection time delay leads to the formation of other phases within the system. This outcome is due to the extremely rapid nature of the reaction between La_2_Se and the present metal precursors, which severely limits any control over the reaction speed. These findings underline that the steric nature of the acyl chain in the selenium precursor and the presence of a thiol ligand dominate in the final NC size, size distribution, and colloidal stability.

The use of bis­(acyl) selenides offers the possibility to obtain AgBi­(S_1–*x*_Se_*x*_)_2_ NCs by mixing the selenide precursors with sulfide-based precursors. AgBiS_2_ and AgBiSe_2_ exhibit similar cubic crystal structures, and alloying on the anion side allows for gradually fine-tuning the bandgap and shifting the spectral response further into the NIR.^37,38^ While mixed silver bismuth sulfide selenide NCs have been reported by Akgul et al.,^26^ the lowest reported selenium contents were 25% and 50%. The easy handling of bis­(acyl) selenides offers the realization of much smaller and more tunable Se/S ratios. To achieve this goal, we selected La_2_Se as the selenium precursor since it provides the narrowest size distribution for the AgBiSe_2_ NCs. Additionally, we utilized bis­(acyl) sulfides as the sulfur precursor. La_2_S is also a colorless, odor-free, and air-stable precursor, which is highly soluble in organic solvents and which was recently used by us and others for the synthesis of sulfide-based NCs.^39^ The AgBi­(S_1–*x*_Se_*x*_)_2_ NCs were synthesized by injecting the desired ratio of sulfur and selenium precursors into the reaction mixture, while the total amount of precursor with respect to the metal ions and the other reaction parameters remained unchanged. While the optical spectroscopy analysis of the resulting and purified NCs shows an intended shift of the absorption edge to longer wavelengths with respect to the rising content of selenium in the system, the corresponding TEM images show that also the size of NCs increases undesirably from 3.1 to 7.5 nm with increasing selenium content in the system (Figure S10). Thus, it remains unclear to what extent the band gap of the resulting NCs is affected by the mixture at the anion site and to what degree an increasing particle size and potential reduced quantum confinement effect are responsible for this observation. Furthermore, the size distribution of the different synthesized NCs is not as narrow as expected and desired (Figure S11), and the TEM images clearly reveal the presence of big and small nanoparticles. The XRD measurements show no significant change in the lattice parameters of AgBi­(S_1–*x*_Se_*x*_)_2_ NCs compared to AgBiS_2_ NCs, other than a change in peak intensity, suggesting that selenium does not get incorporated into the crystal lattice upon addition (Figure S12). However, taking the TEM analysis into account, using the La_2_S/La_2_Se mixture, we cannot fully exclude the presence of separate AgBiS_2_, AgBiSe_2_, alongside AgBi­(S_1–*x*_Se_*x*_)_2_ nanoparticles, especially for 30% and 40% selenium content. We believe these results to be a direct consequence of a varying reactivity of the S- and Se-precursors—in particular, La_2_Se seems to react significantly faster than La_2_S under the given reaction condition, due to the weaker (C=O)–Se bond, which is a consequence of the larger atomic radius and higher polarizability of selenium. Such differences could result in selective growth of NCs with increasing Se content and small phase-separated AgBiS_2_ NCs, as observed in the TEM images. The difference in reactivity between analogous sulfur and selenium precursors is often observed in the colloidal synthesis of metal chalcogenide nanocrystals. To avoid this in syntheses involving the simultaneous injection of sulfur and selenium precursors, compounds with different molecular structures are used to balance their reactivities and achieve controlled incorporation of both chalcogens.^40^

In order to compensate for the high reactivity of La_2_Se, we were aiming to implement a sulfur precursor that demonstrates a similarly high reaction rate for synthesizing truly alloyed AgBi­(S_1–*x*_Se_*x*_)_2_ NCs with different S/Se ratios. For this, we utilized hexamethyldisilathiane, more commonly abbreviated as (TMS)_2_S. This commercially available, highly reactive, and volatile sulfur precursor is routinely used as a sulfur precursor in the hot-injection synthesis of AgBiS_2_ NCs.^4,6,7^ Our experiments show that (TMS)_2_S/La_2_Se mixtures allow the successful synthesis of alloyed AgBi­(S_1–*x*_Se_*x*_)_2_ NCs with a selenium content of 0 to 50% Se (*x* = 0–0.5). The TEM images of the synthesized and purified NCs show a uniform size, with an average of around 3.3 nm, in conjunction with a narrow size distribution independent of the selenium content (Figure 2). In all cases, high-resolution TEM images confirm the formation of crystalline NCs rather than amorphous particles. For higher selenium contents, the NC size and their tendency to become more irregularly shaped increases and a shift to lower angles for increasing concentration of selenium can be observed (Figures S13 and S14). The resulting AgBi­(S_1–*x*_Se_*x*_)_2_ NCs demonstrate colloidal stability for several weeks or months due to stabilization with 1-DDT. Interestingly, further experiments showed that the synthesized NCs can show colloidal stability up to a certain percentage of Se content (20%) without the addition of 1-DDT during the hot-injection process, which can be attributed to the comparatively low selenium content in these samples. Further analysis with scanning transmission electron microscopy energy-dispersive X-ray spectroscopy (STEM–EDX) of the (TMS)_2_S/La_2_Se-based AgBi­(S_1–*x*_Se_*x*_)_2_ NCs was conducted in order to confirm the alloying of the NCs. The elemental mapping distinctly depicts the presence of both sulfur and selenium homogeneously in all relevant NCs, along with silver and bismuth (Figure S15), which can be seen in the analyzed areas in Figure 2. In more detail, we evaluate the background-subtracted EDX intensities locally, as indicated by the vertical yellow arrows in Figure S16a. The corresponding horizontal direction-averaged profiles are compiled in Figure S16b, showing the intensity distribution for silver (gray), sulfur (blue), and selenium (red). The peaks of the silver signal directly identify the particle locations within the profiles and coincide with increased signals for sulfur and selenium in most cases. Remarkably, as the selenium and sulfur signals increase proportionally, the hypothesis of a possible phase separation related to these two elements cannot be supported by this investigation. These findings rather suggest a homogeneous alloying of sulfur and selenium within the NCs, instead. The increased sulfur background in between the particles is a consequence of sulfur-containing precursor residuals distributed across the supporting carbon film. The results highlight that indeed alloyed NCs are formed and not a mixture of phase-separated AgBiS_2_ and AgBiSe_2_ NCs. This confirms the success of our synthetic approach and underlines the potential of bis­(acyl) selenides to fine-tune the anion composition in such nanomaterials.

**Figure 2:**
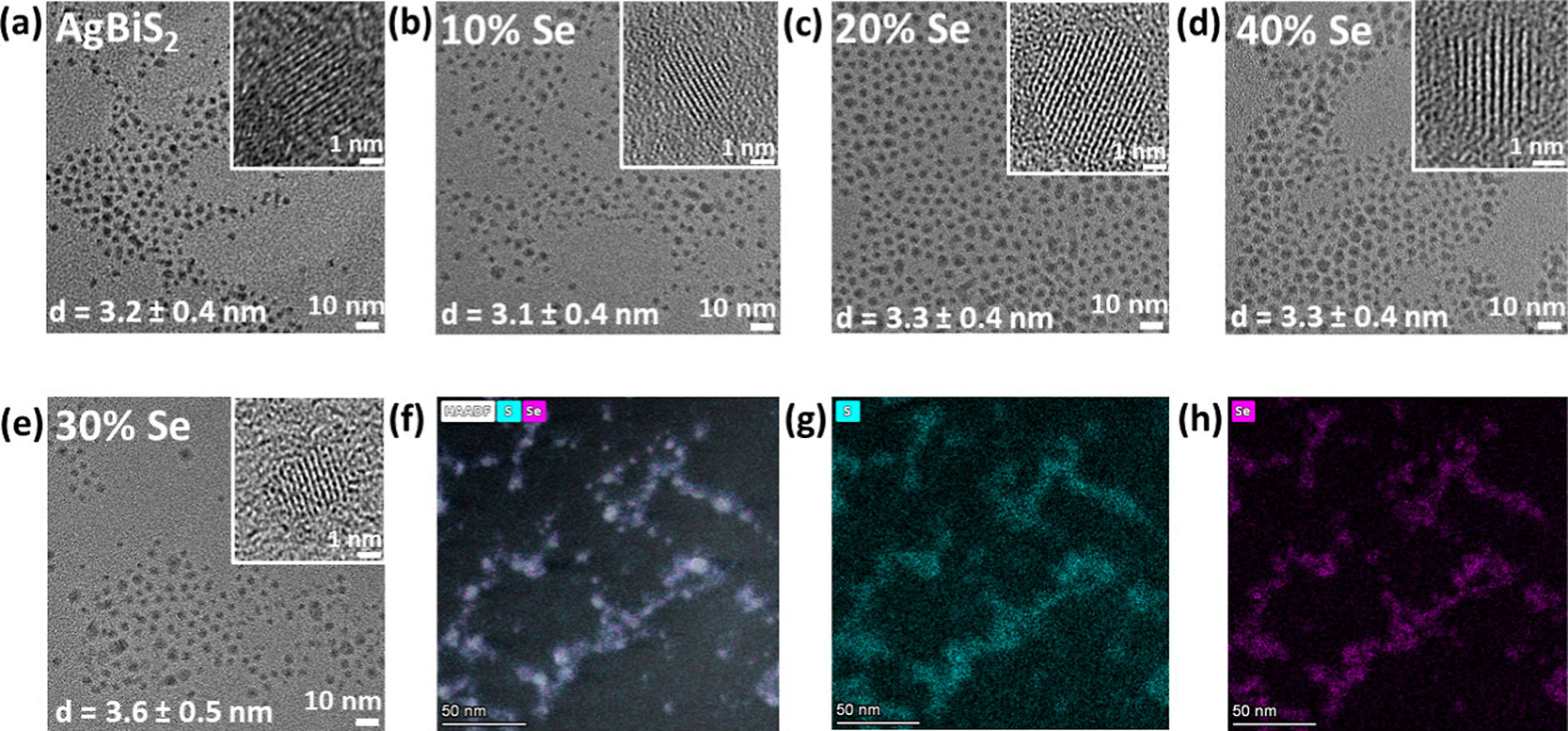
Characterization of AgBiS_2_ and AgBi­(S_1–*x*_Se_*x*_)_2_ NCs, synthesized with (TMS)_2_S and (TMS)_2_S/La_2_Se mixtures, respectively, with varying S/Se ratios (intended Se content given in %). (a) TEM image of synthesized AgBiS_2_ NCs. TEM image and HRTEM inset of AgBi­(S_1–*x*_Se_*x*_)_2_ NCs with (b) 10%, (c) 20%, (d) 40%, and (e) 30% Se content. (f) HAADF-STEM image of NCs, depicting S and Se within the system. (g) Composite EDX map of S. (h) Composite EDX map of Se.

Structural analysis provides further insight into the compositions of the AgBi­(S_1–*x*_Se_*x*_)_2_ NCs depending on the selenium content, as shown on Figure 3. By judging the XRD patterns, it is apparent that not only does the (111) reflection at around 26° shift with increasing selenium content in the NCs toward smaller angles but also it shows a decrease in intensity with increasing selenium content. Additionally, the second prominent (200) reflection in the XRD patterns at around 31° shows a shift toward 30° (Figure 3b). Figure 3c illustrates the shift in peak positions for the three most prominent reflections, in line with the incorporation of the bigger selenium in the NC lattice. In all characterized NCs synthesized with (TMS)_2_S/La_2_Se mixtures, we observe small changes of the *d*-spacing values with the implementation of larger amounts of selenium. Interplanar distances of pure synthesized AgBiS_2_ and AgBiSe_2_ compounds show good correspondence with values noted in literature,^36^ while the determined *d*-spacings of the alloyed NCs are in-between the values of the pure compounds. Like in the case of La_2_S/La_2_Se mixtures, the XRD patterns of the (TMS)_2_S/La_2_Se precursor mixtures do not exhibit any reflections from phase-separated materials. Taken together, the clean XRD patterns and shift in peak position additionally confirm the formation of alloyed AgBi­(S_1–*x*_Se_*x*_)_2_ NCs.

**Figure 3:**
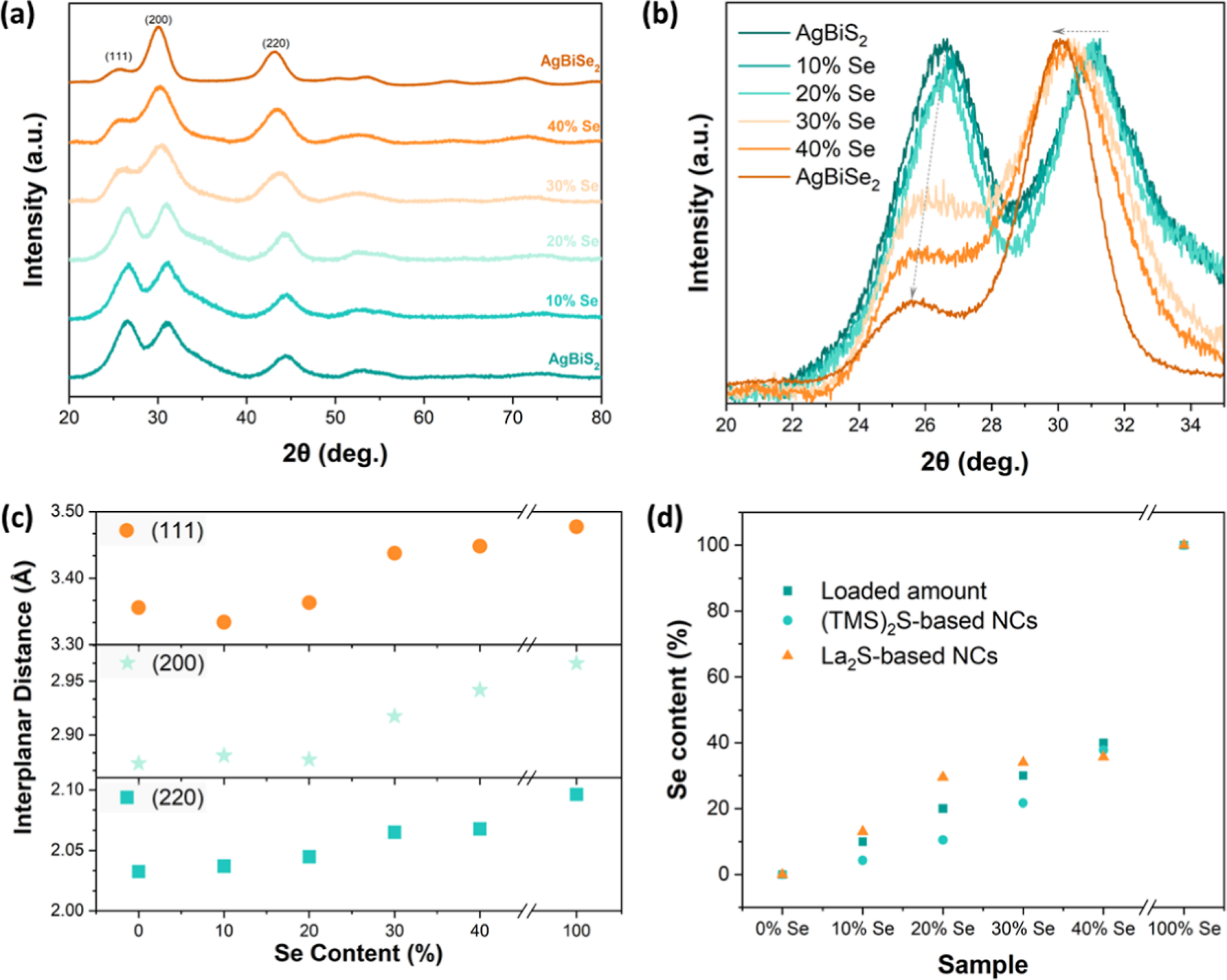
Structural analysis of (TMS)_2_S NCs. (a) XRD pattern of synthesized AgBi­(S_1–*x*_Se_*x*_)_2_ NCs with varying contents of selenium. (b) Magnified diffraction pattern for the (111) and (200) reflections with increasing Se content. (c) Extracted peak positions for each NC sample. (d) ICP-OES composition of (TMS)_2_S- and La_2_S-based AgBi­(S_1–*x*_Se_*x*_)_2_ NCs.

Inductively coupled plasma optical emission spectroscopy (ICP-OES) was conducted in order to precisely determine the composition of the synthesized NCs with respect to the loaded amounts of reagents (Figure 3d). With this analysis technique, we can precisely determine the composition of the synthesized AgBi­(S_1–*x*_Se_*x*_)_2_ NCs. According to the measurement results, the Se/S ratio increases as intended by the mixing of precursors. The (TMS)_2_S/La_2_Se-based NCs generally exhibit a consistent trend, which can also serve as an indication of the formation of an alloyed system. Moreover, the La_2_S/La_2_Se-based alloyed NCs show a more scattered Se/S ratio with respect to the loaded amounts, which can further confirm the difference in reactivity between the La_2_S and La_2_Se precursors and the formation of phase-separated NCs. The ICP-OES analysis value results can be found in Tables S1 and S2.

The uniform NC size and monotonic increase in the Se/S ratio allow for a reliable extraction of a composition-dependent band gap for the AgBi­(S_1–*x*_Se_*x*_)_2_ NCs. The optical absorbance spectra of the AgBi­(S_1–*x*_Se_*x*_)_2_ NCs are depicted in Figure 4a. The spectra are only slightly red-shifted, indicative of the tuning of the band gap depending on the alloy composition. Band gap analysis utilizing Tauc plots (Figure 4b) shows a narrowing of the optical bandgap with increasing selenium content. The calculated values based on the Tauc plot are 1.27, 1.17, 1.03, 0.98, 0.95, and 0.92 eV for AgBiS_2_, respectively (Figure 4c). This precisely shows the fine-tuning of the band gap with the implementation of different contents of selenium into the NC system. The resulting AgBi­(S_1–*x*_Se_*x*_)_2_ NC inks demonstrate excellent colloidal stability for several weeks of storage as shown in Figures S17 and S18 due to stabilization with 1-DDT. To further confirm the colloidal stability of the NCs, dynamic light scattering (DLS) measurements were performed first on a fresh sample and, afterward, 7 days later on the same sample (Figure S19). As shown, the mean NC particle size is maintained even after prolonged storage under ambient conditions, enabling the use and study of these NC dispersions for solution-processed optoelectronics.

**Figure 4:**
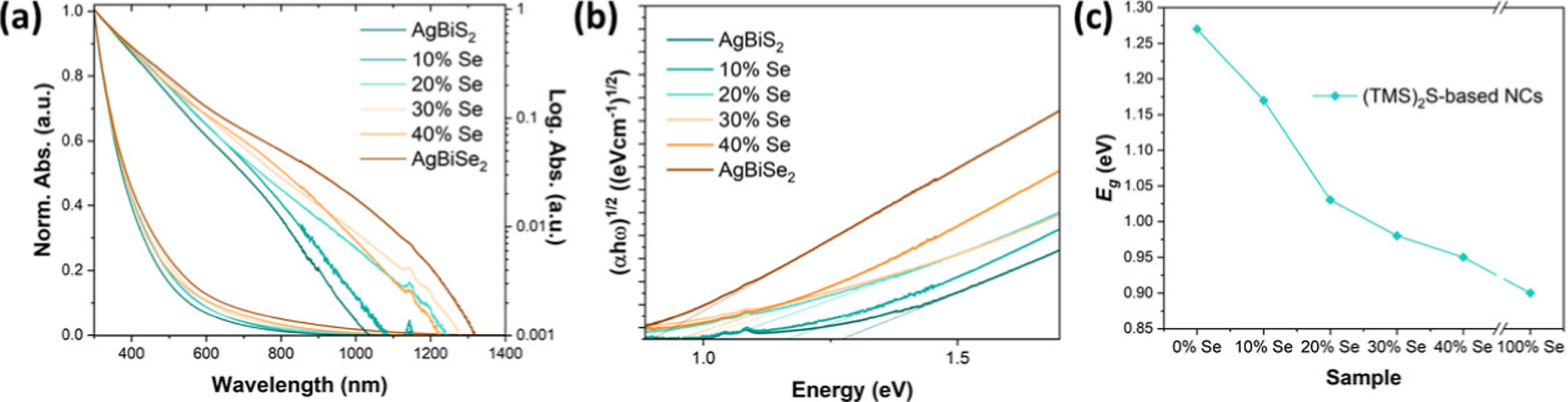
Characterization of (TMS)_2_S-based AgBi­(S_1–*x*_Se_*x*_)_2_ NCs. (a) Optical absorbance spectra of AgBi­(S_1–*x*_Se_*x*_)_2_ NCs, normalized and plotted in linear and logarithmic scales. (b) Band gap analysis with a Tauc plot of AgBi­(S_1–*x*_Se_*x*_)_2_ NCs. (c) Calculated band gap *E*_g_ values for NCs.

## Conclusions

In our study, we demonstrate the successful hot-injection synthesis of colloidally stable ternary AgBiSe_2_ and quaternary AgBi­(S_1–*x*_Se_*x*_)_2_ NCs with narrow size distributions by utilizing bis­(acyl) selenide precursors. These air-stable, solid selenium precursors not only simplify handling and processing under ambient conditions, but also allow fine-tuning of the sulfur-to-selenium ratio in silver bismuth chalcogenide NCs, enabling precise control of the optical band gap without compromising the size, size distribution, and crystallinity of the NCs. The structural, compositional, and optical properties of the resulting quaternary AgBi­(S_1–*x*_Se_*x*_)_2_ NCs prove that these nanocrystals are alloyed structures. Our synthetic approach unlocks the possibility to finely tune the optoelectronic properties of environmentally friendly and earth-abundant silver bismuth chalcogenide NCs for future application in optoelectronic devices, such as photovoltaics and NIR photodetectors.

## Experimental Section

### Chemicals

Silver acetate (Ag­(OAc), anhydrous, 99%), bismuth­(III) acetate (Bi­(OAc)_3_, 99.999%), toluene (anhydrous, 99.8%), 1-octadecene (90%), 1-dodecanethiol (98%), and tetrahydrofuran (THF, 99.8%, anhydrous) were purchased from Thermo Fisher Scientific. Hexamethyldisilane (synthesis grade), sulfur powder (S, 99.98%), and selenium powder (Se, 99.5%) were purchased from Sigma-Aldrich. Diethyl ether (99.5%), sodium chloride (NaCl, 99.5%), sodium sulfate (99%, anhydrous), acetone (99.8%), and lithium aluminum hydride (LiAlH_4_, 2.4 M in THF) were purchased from Fisher Chemical. Tetrachloroethylene (99.0%) was purchased from Merck. Oleic acid (99%) was purchased from Acbr. Nitric acid (p.a.) and hydrogen peroxide (>30%) were purchased from CHEMSOLUTE. Lauroyl chloride (98.0%), stearoyl chloride (96.0%), and benzoyl chloride (98.0%) were purchased from TCI.

### Synthesis of Bis­(acyl) Chalcogenides

Bis­(stearoyl) selenide (St_2_Se), bis­(benzoyl) selenide (Bz_2_Se), and bis­(lauroyl) sulfide (La_2_S) were synthesized using published protocols.^32,39^ For the synthesis of bis­(lauryol) selenide (La_2_Se), we adopted the protocol for St_2_Se. Briefly, selenium powder (3.356 g, 42.5 mmol) was added to 250 mL of anhydrous THF under an inert atmosphere. The obtained suspension was cooled down to −10 °C, and then lauroyl chloride (33.3 mL, 141.6 mmol) was added dropwise under vigorous stirring. The reaction mixture was stirred for 2 h in a nitrogen atmosphere at −10 °C, then quenched with 10 mL of H_2_O, and diluted with 350 mL of diethyl ether. The organic phase was washed with 300 mL of saturated NaCl solution five times, dried over Na_2_SO_4_, filtered, and evaporated under reduced pressure until approximately 70 mL of solution remained. The product was crystallized in a refrigerator (3 °C) overnight. The crystallized product was separated by vacuum filtration, washed with ice-cold diethyl ether, and dried under vacuum. It was recrystallized twice, yielding La_2_Se colorless powder.

### Synthesis of AgBiS_2_ NCs

0.8 mmol of Ag­(OAc) and 1 mmol of Bi­(OAc)_3_ were loaded into a three-neck round-bottom flask with 4 mL of ODE and 6 mL of OA. The mixture was purged and heated to 100 °C for 1 h under vacuum in order to remove air and water. Afterward, the reaction flask was filled with N_2_, and 1 mmol of La_2_S or (TMS)_2_S dissolved in toluene or ODE was swiftly injected into the mixture. Upon injection, the reaction mixture color changes from clear to dark brown. In the case of La_2_S, the reaction mixture was left to cool down to room temperature gradually, while the mixture with (TMS)_2_S was cooled rapidly with a water bath. The obtained NCs were precipitated by the addition of an appropriate amount of acetone and centrifugation. The supernatant was discarded, and the precipitate was dispersed in toluene. This process was repeated twice, and the final supernatant was dispersed in toluene and filtered through a PTFE filter (pore size 0.22 μm) for further storage and use.

### Synthesis of AgBiSe_2_ NCs

The process is similar to that of the previously described synthesis of AgBiS_2_ NCs. Instead of a sulfur precursor, 1 mmol of La_2_Se (or an alternative selenium precursor) and 0.7 mL of 1-DDT were simultaneously injected into the reaction mixture and rapidly cooled down to room temperature with a water bath. The precipitation and redispersion are analogous to those of AgBiS_2_ NCs.

### Synthesis of AgBi­(S_1–*x*_Se_*x*_)_2_ NCs

The process is similar to that of the described synthesis of AgBiS_2_ NCs above. A mixture of sulfur (La_2_S or (TMS)_2_S) and selenium precursors (La_2_Se) in toluene, along with 1-DDT, was simultaneously injected into the reaction mixture and rapidly cooled down to room temperature with a water bath. The precipitation and redispersion are analogous to that of AgBiS_2_ NCs.

### Characterization

#### X-ray Diffraction (XRD)

XRD patterns were recorded using a Rigaku SmartLab powder X-ray diffractometer, equipped with a HyPix 3000 2D X-ray detector in a parallel beam setup in θ–2θ mode. A Cu Kα rotating anode was used as the X-ray source. Measurements were performed in ambient conditions.

#### UV/Vis Spectroscopy

A Jasco V-770 spectrometer was used in order to measure the optical absorption spectra in the ultraviolet–visible and the near-infrared regions. Absorbance measurements were carried out in 1 cm wide quartz cuvettes.

#### Inductively Coupled Plasma-Optical Emission Spectroscopy (ICP-OES)

ICP-OES measurements were performed by using a Thermo Fisher Scientific iCAP 6500 Duo View. The NCs were solved with nitric acid and hydrogen peroxide and filled to 15 mL. The elemental concentration for each element was measured at different wavelengths, and an average of the result at different wavelengths per element was calculated. Each measurement was performed three times, and the average of the results was calculated.

#### Transmission Electron Microscopy (TEM)

HRTEM images were acquired at 300 kV acceleration voltage using a Thermo Fisher Titan^3^ 80-300 double-aberration-corrected transmission electron microscope equipped with a Gatan OneView camera, model 1095. Particle sizes were determined by taking several diameter measurements in ImageJ per particle and calculating the average.

#### High-Angle Annular Dark-Field Scanning Transmission Electron Microscopy (HAADF-STEM)

HAADF-STEM was performed on a Thermo Fisher Talos F200i operating at 200 kV acceleration voltage using a semiconvergence angle of 10.5 mrad and a collection angle range of 32–192 mrad. **Energy dispersive X-ray spectroscopy (EDX) measurements** using a Dual-X detection system were realized by scanning a 204 × 206 mm^2^ area at a step size of 0.5 nm with a probe current of about 1.5 nA and a dwell time of 10 μs. The elemental maps were extracted from these measurements by using background-subtracted intensities of the Bi Lα, Ag Lα, S Kα, and Se Kα lines, summed over 1307 frames. To reduce contamination during scanning, the sample was Ar/O plasma cleaned for 20 s at a flow rate of 30 sccm before STEM measurements.

#### Dynamic Light Scattering (DLS)

The hydrodynamic sizes of the NCs were investigated with a Zetasizer Ultra (Model Ultra Red from Malvern Instruments) at a wavelength of 633 nm, using a He–Ne laser at a scattering angle of 90°. Samples were diluted 10 times before the measurements.

## Supplementary Material



## Data Availability

Data is plotted and provided within figures and graphs and in the Supporting Information. All data is available from the authors upon request.

## Notes

ATR FTIR spectrum of bis­(lauroyl) selenide powder, ^1^H-NMR and ^13^C-NMR spectra of bis­(lauroyl) selenide, stacked ^13^C-NMR spectra of freshly synthesized La_2_Se and La_2_Se after storage in a sealed vial under ambient air for 2 months, AgBiSe_2_ NCs, synthesized without the presence of 1-DDT , particle size distributions of AgBiSe_2_ NCs, synthesized using different precursors, characteristics of synthesized AgBiSe_2_ NCs grown over time at a higher temperature with various time delays of 1-DDT injection, characterization of AgBiS_2_ and AgBi­(S_1–*x*_Se_*x*_)_2_ NCs, synthesized with La_2_S and La_2_S/La_2_Se mixtures with varying ratios of S/Se, particle size distributions, structural analysis, particle size distributions of (TMS)_2_S-based AgBiS_2_ and AgBi­(S_1–*x*_Se_*x*_)_2_ NCs with different Se contents, characterization of (TMS)_2_S-based AgBi­(S_1–*x*_Se_*x*_)_2_ NCs, EDX characterization and elemental mapping of (TMS)_2_S-based AgBi­(S_1–*x*_Se_*x*_)_2_ NCs with 30% selenium content, synthesized (TMS)_2_S-based AgBi­(S_1–*x*_Se_*x*_)_2_ NCs with different selenium contents, fresh and several weeks after storage, DLS measurements of (TMS)_2_S-based AgBi­(S_1–*x*_Se_*x*_)_2_ NCs with 30% selenium content, freshly synthesized and aged for 7 days, and ICP-OES analysis results of (TMS)_2_S-based and La_2_S-based AgBi­(S_1–*x*_Se_*x*_)_2_ NCs (PDF)
