# Supplementary material for: Colloidal AgBiSe2 and AgBi(S1–x Se x )2 Nanocrystals with Composition-Tunable Properties Based on Bis(acyl) Selenide Precursors
Source: ACS Omega. 2026 Jul 21;11(30):45297–305. doi: 10.1021/acsomega.6c04337 (PMC13448908; doi:10.1021/acsomega.6c04337)
Supplement: Supplementary file 1 [file ao6c04337_si_001.pdf]

Supporting information

Colloidal $AgBiSe_2$ and $AgBi(S_{1-x}Se_x)_2$ nanocrystals with Composition-tunable Properties Based on Bis(acyl) selenide Precursors

Julia Prodan[1], Anatol Prudnikau[1], Falk Röder[1,2], Xuan Qi[1], Fabian Paulus[1,*]

[1] Leibniz Institute for Solid State and Materials Research (IFW) Dresden, Helmholtzstraße 20, 01069 Dresden, Germany

[2] Leibniz Institute of Polymer Research (IPF) Dresden, Hohe Straße 6, 01069 Dresden, Germany

* corresponding author: f.paulus@ifw-dresden.de

Table of Contents

Figure S1. ATR FTIR spectrum of bis(lauroyl) selenide powder. 3

Figure S2. ^{1}H-NMR (a) and (b) ^{13}C-NMR spectra of bis(lauroyl) selenide. Spectra were recorded in $CDCl_3$ on a Bruker Avance II 500 MHz spectrometer. 4

Figure S3. Stacked ^{13}C NMR spectra of freshly synthesized La_2Se and La_2Se after storage in a sealed vial under ambient air for 2 months. 5

Figure S4. $AgBiSe_2$ NCs, synthesized without the presence of 1-DDT. 5

Figure S6. Characteristics of synthesized $AgBiSe_2$ NCs grown over time. 6

Figure S7. Characteristics of $AgBiSe_2$ NCs, synthesized at a higher temperature. 7

Figure S8. TEM images of $AgBiSe_2$ NCs with various time delays of 1-DDT injection into the synthesis mixture in an attempt to grow NCs of different sizes. 7

Figure S9. Characterization of $AgBiSe_2$ NCs with various time delays of 1-DDT injection into the synthesis mixture. 8

Figure S10. Characterization of $AgBiS_2$ and $AgBi(S_{1-x}Se_x)_2$ NCs, synthesized with La_2S and La_2S:La_2Se mixtures respectively, with varying ratios of S:Se. 8

Figure S11. Particle size distributions of La_2S-based $AgBiS_2$ and $AgBi(S_{1-x}Se_x)_2$ NCs with different Se contents. 9

Figure S12. Structural analysis of La_2S-based NCs. (a) XRD patterns of synthesized $AgBi(S_{1-x}Se_x)_2$ NCs with varying contents of selenium. 9

Figure S13. (a) Particle size distributions of $(TMS)_2S$-based $AgBiS_2$ and $AgBi(S_{1-x}Se_x)_2$ NCs with different Se contents, up to 40% selenium content. (b) TEM images and particle size distributions of NCs with 50-70% selenium content. 10

Figure S15. Characterization of $(TMS)_2S$-based $AgBi(S_{1-x}Se_x)_2$ NCs with 30% selenium content. 11

Figure S16. (a) EDX elemental map as composite images of Se (magenta) and S (cyan) background subtracted intensities. (b) Vertical line profiles as indicated in (a) showing the horizontally over 25nm averaged intensities for Se (red), S (blue) and Ag (gray). 11

Figure S17. Synthesized $(TMS)_2S$-based $AgBi(S_{1-x}Se_x)_2$ NCs with different selenium contents, stabilized with 1-DDT. 12

Figure S19. Dynamic light scattering (DLS) measurements of $(TMS)_2S$-based $AgBi(S_{1-x}Se_x)_2$ NCs with 30% selenium content, both freshly synthesized and aged for 7 days. 13

Table S1. ICP-OES analysis results of $(TMS)_2S$-based $AgBi(S_{1-x}Se_x)_2$ alloyed NCs, given in µg per 15 ml of solution. 13

Table S2. ICP-OES analysis results of La_2S-based $AgBi(S_{1-x}Se_x)_2$ NCs, given in µg per 15 ml of solution. 13

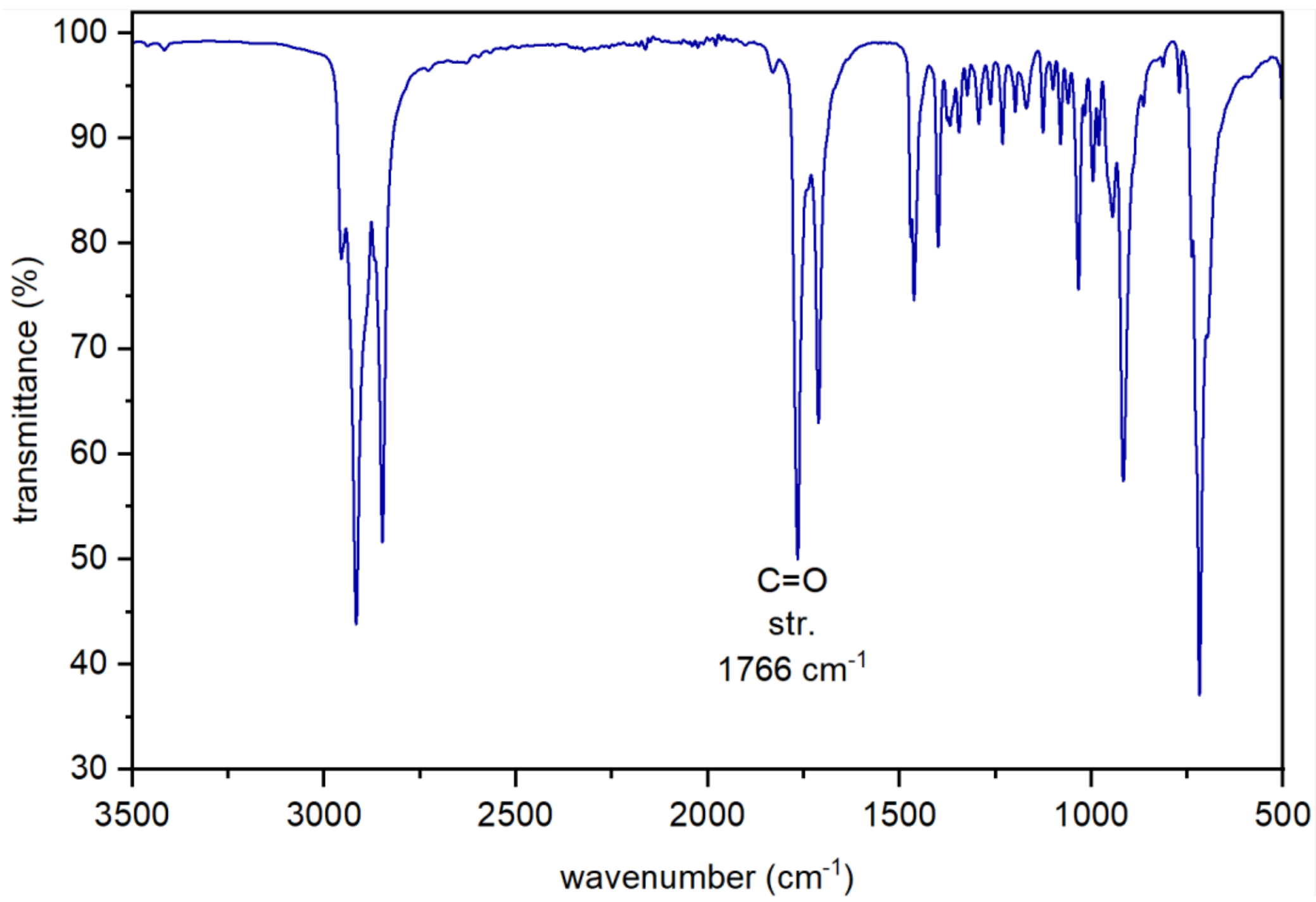

Figure S1. ATR FTIR spectrum of bis(lauroyl) selenide powder.

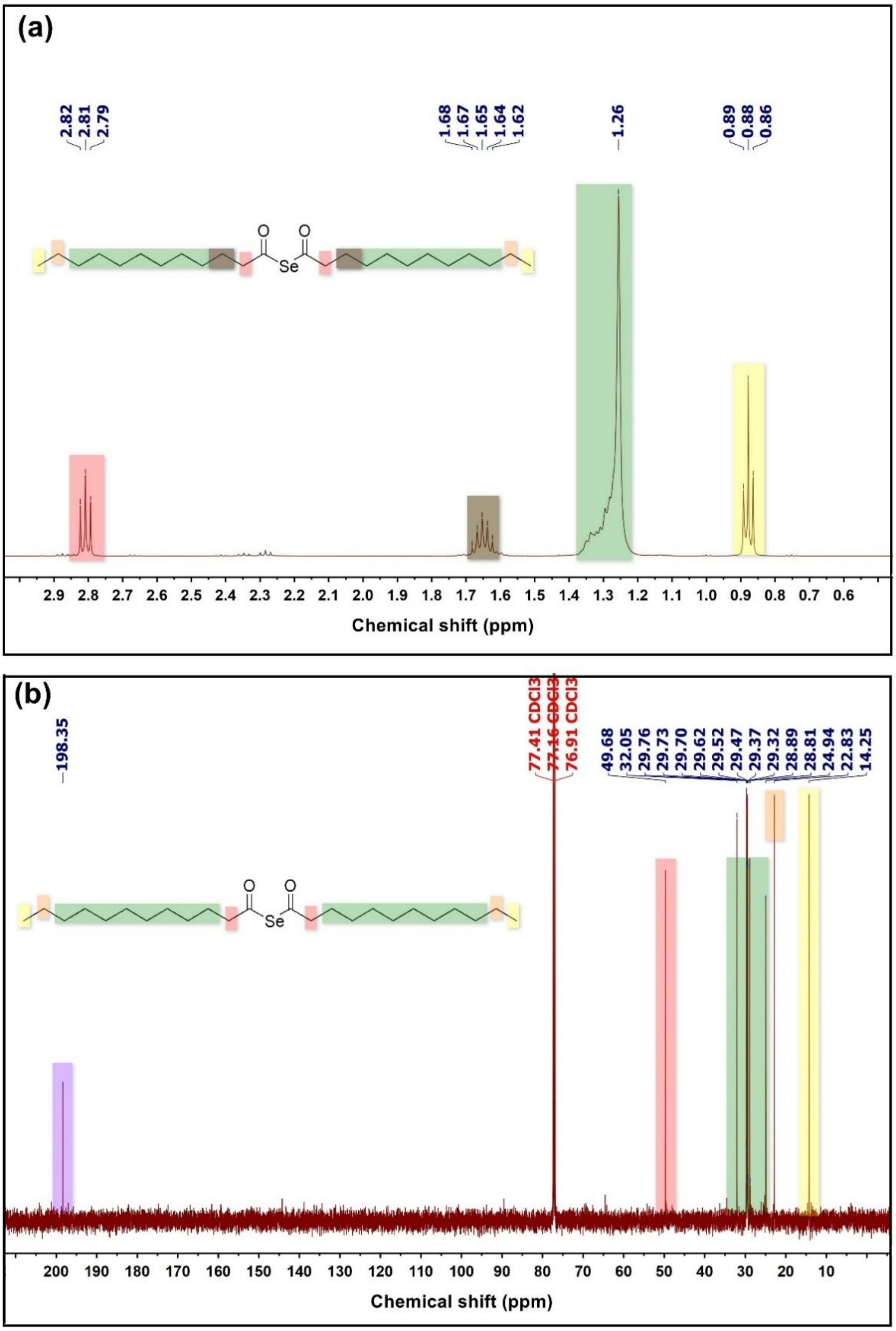

Figure S2. ^{1}H-NMR (a) and (b) ^{13}C-NMR spectra of bis(lauroyl) selenide. Spectra were recorded in $CDCl_3$ on a Bruker Avance II 500 MHz spectrometer.

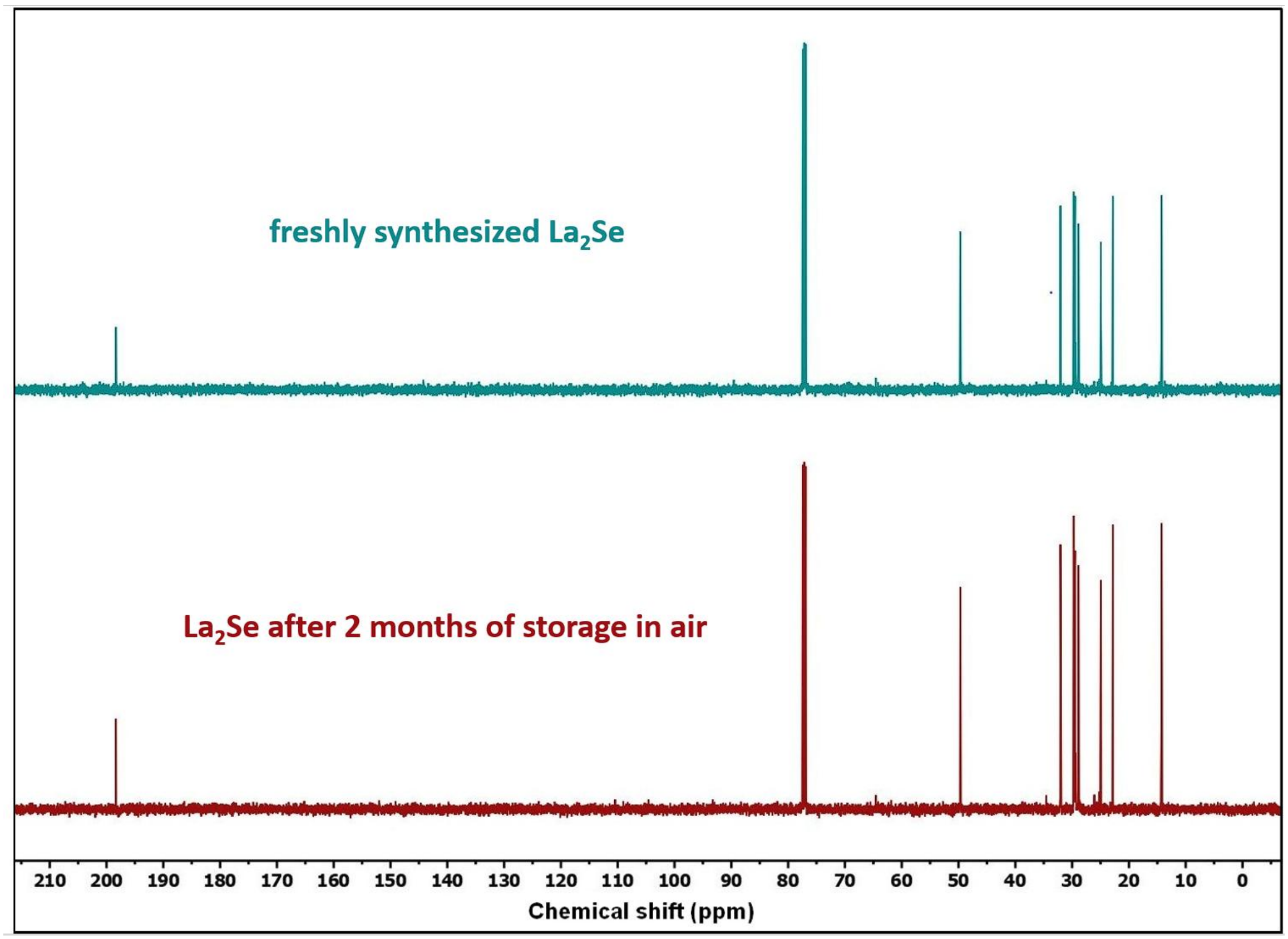

Figure S3. Stacked ^{13}C NMR spectra of freshly synthesized La_2Se and La_2Se after storage in a sealed vial under ambient air for 2 months.

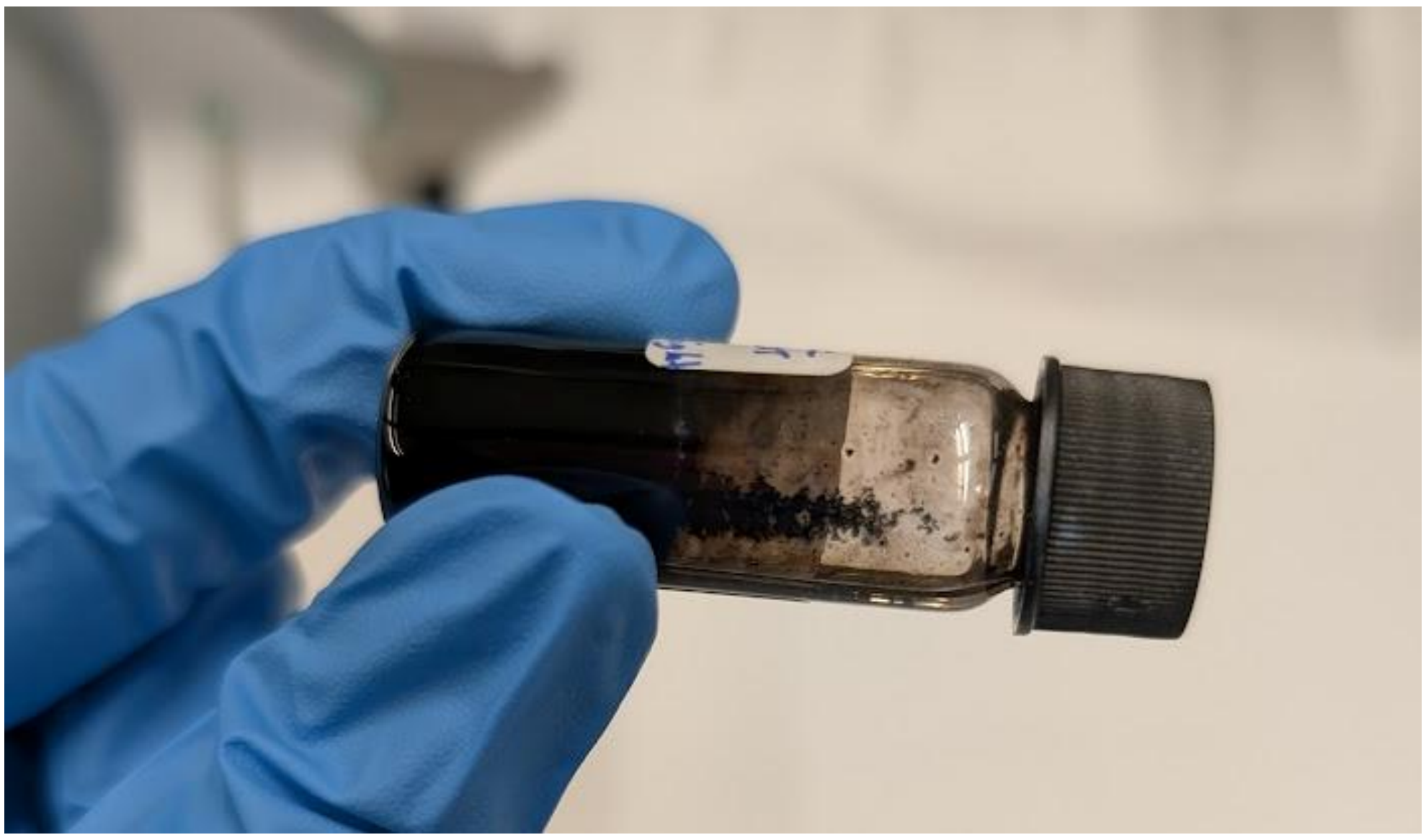

Figure S4. $AgBiSe_2$ NCs, synthesized without the presence of 1-DDT.

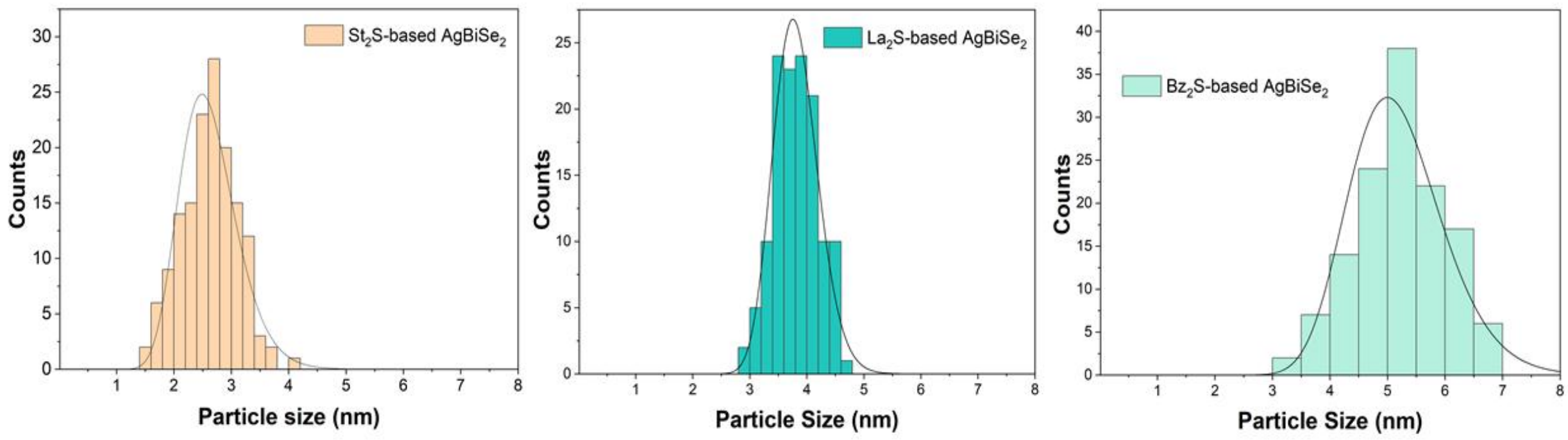

Figure S5. Particle size distributions of $AgBiSe_2$ NCs, synthesized using different precursors.

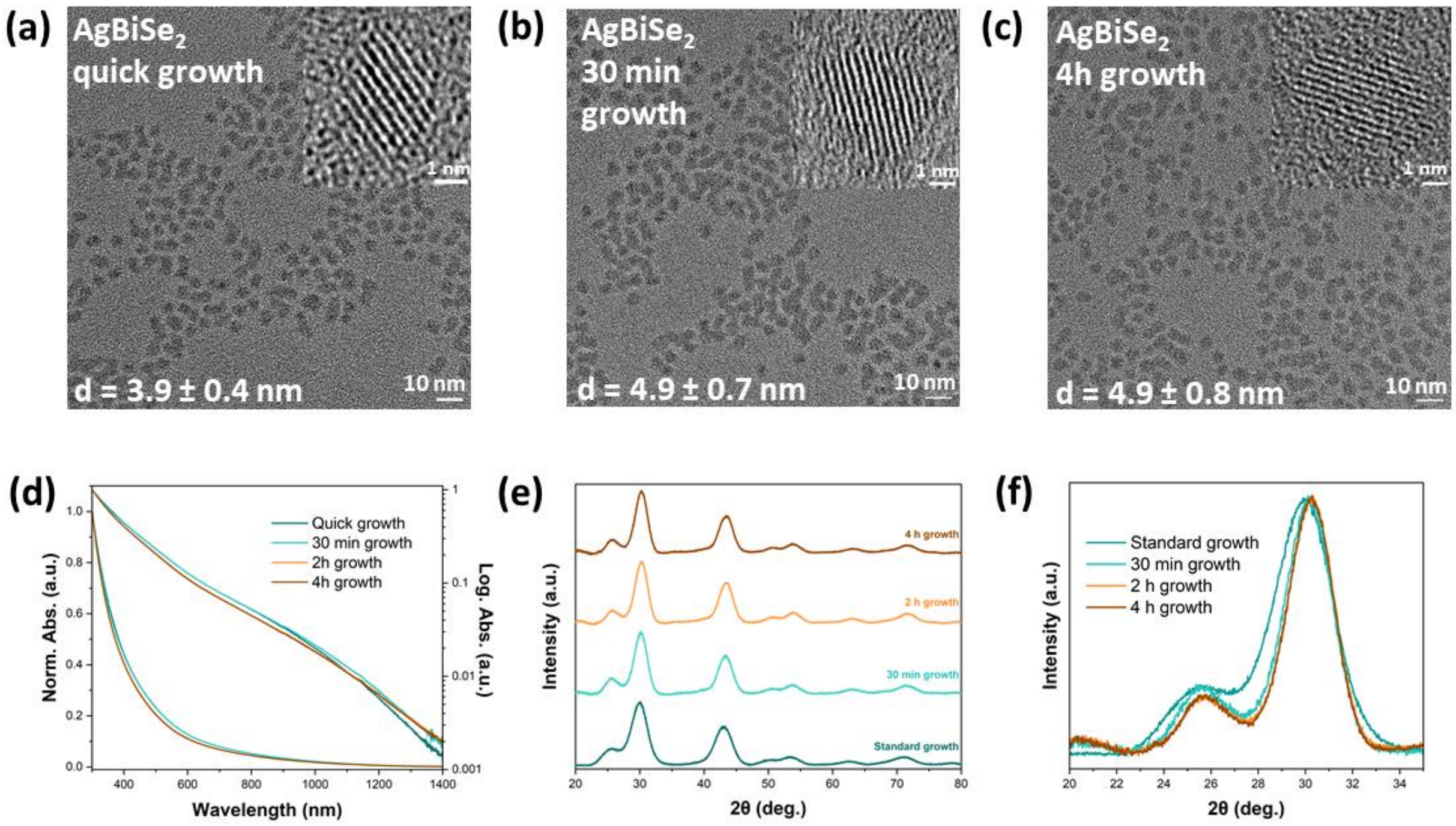

Figure S6. Characteristics of synthesized $AgBiSe_2$ NCs grown over time. (a) TEM image of NCs grown quickly. (b) TEM image of NCs grown over 30 minutes. (c) TEM image of NCs grown over 4 hours. (d) UV/Vis spectra of NCs grown over various amounts of time. (e) Structural analysis of NCs growth over various periods of time. (f) Magnified XRD patterns, depicting the changes in the two most prominent XRD peaks of NCs grown over various periods of time. The $AgBiSe_2$ NCs do not grow in size with time.

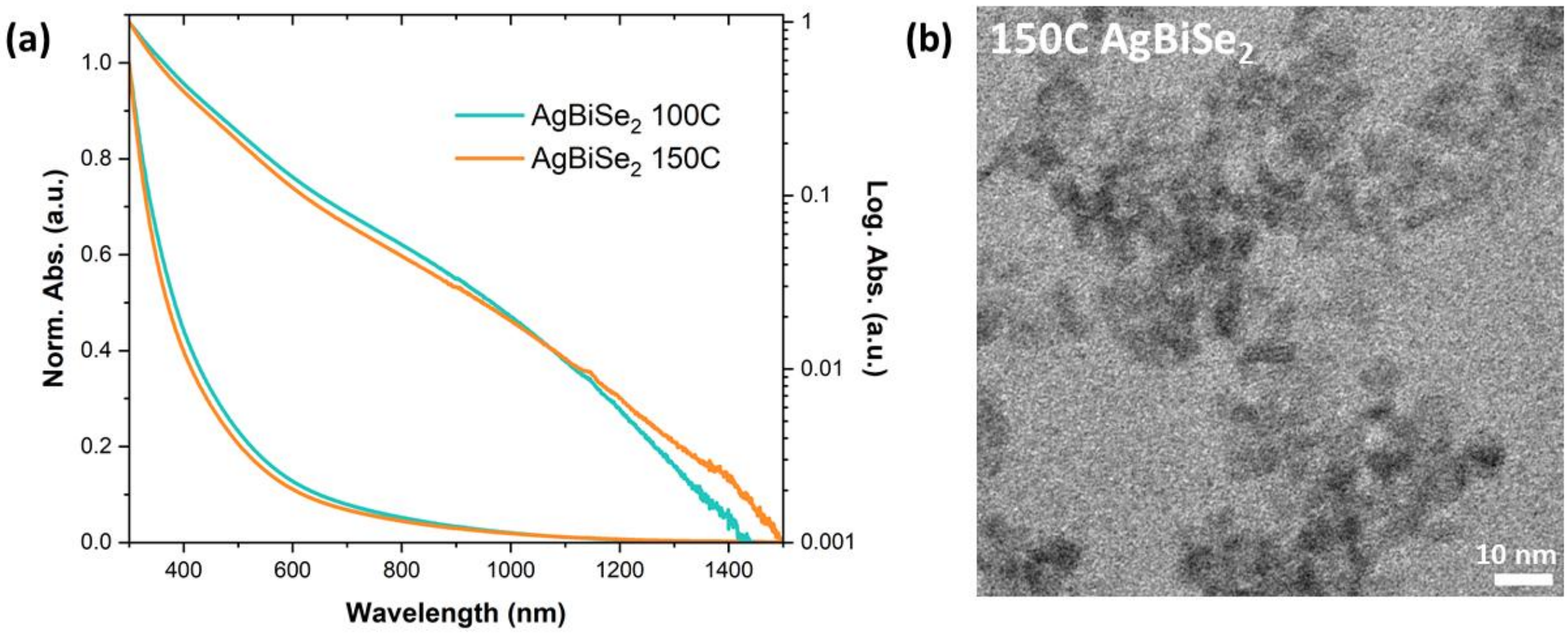

Figure S7. Characteristics of $AgBiSe_2$ NCs, synthesized at a higher temperature. (a) UV/Vis spectra of NCs synthesized at 100°C and 150°C. (b) TEM image of NCs synthesized at 150°C. Attempting to influence the NC particle size with higher temperatures was not possible – the NCs showed aggregation directly after the precursor injection at higher temperatures.

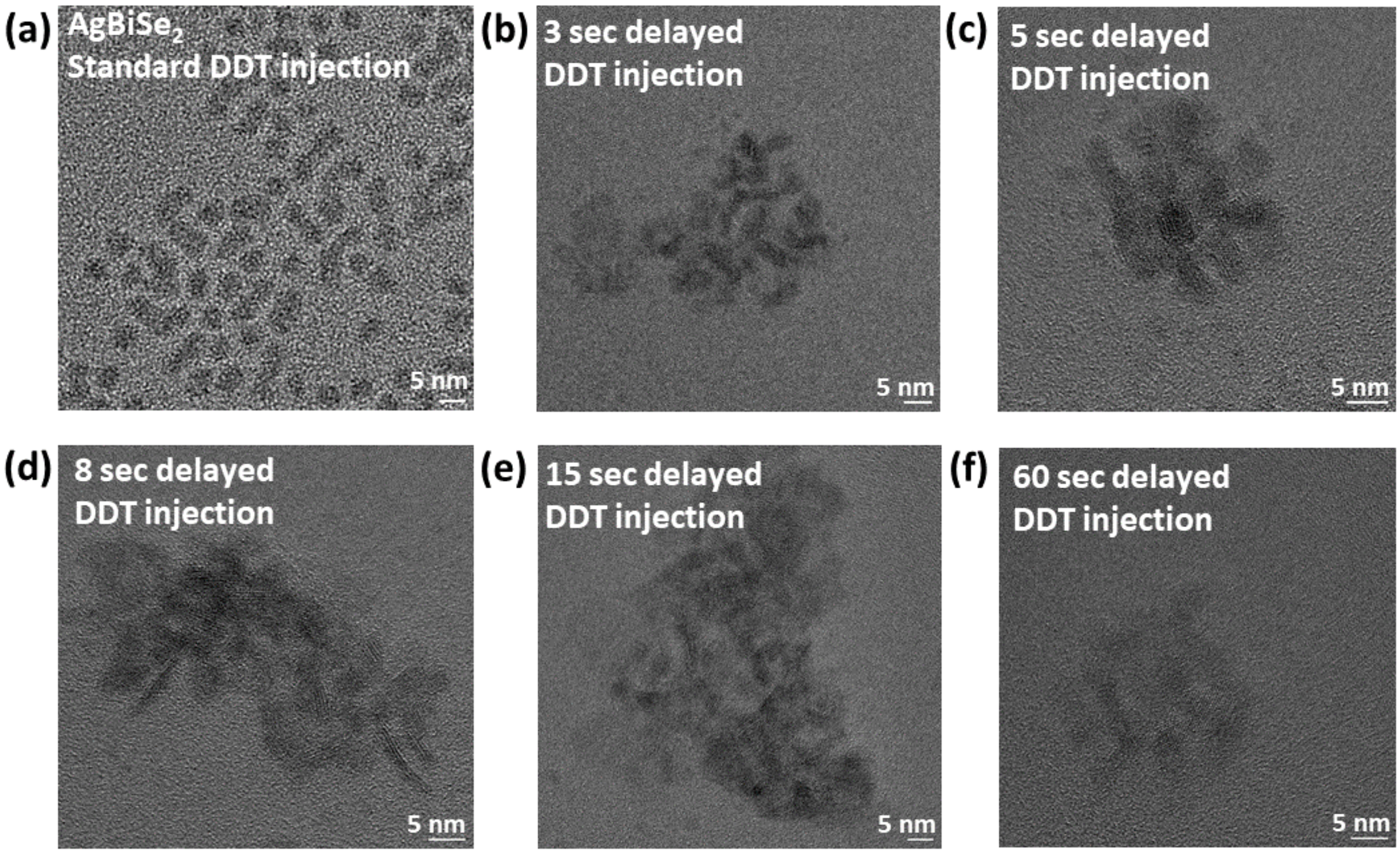

Figure S8. TEM images of $AgBiSe_2$ NCs with various time delays of 1-DDT injection into the synthesis mixture in an attempt to grow NCs of different sizes. (a) Standard injection time. (b) Injection time delayed by 3 seconds. (c) Injection time delayed by 5 seconds. (d) Injection time

delayed by 8 seconds. (e) Injection time delayed by 15 seconds. (f) Injection time delayed by 60 seconds.

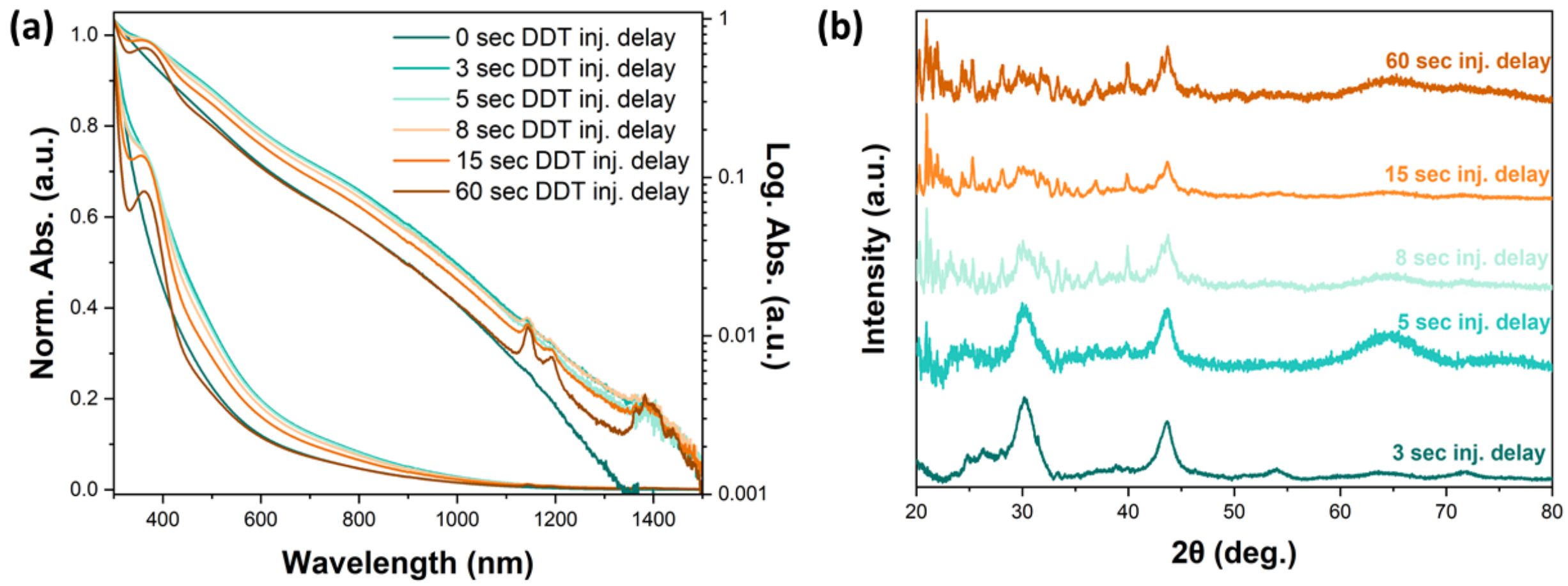

Figure S9. Characterization of $AgBiSe_2$ NCs with various time delays of 1-DDT injection into the synthesis mixture. (a) Optical absorbance spectra of the NCs, plotted in linear and logarithmic scales. (b) XRD patterns of the NCs.

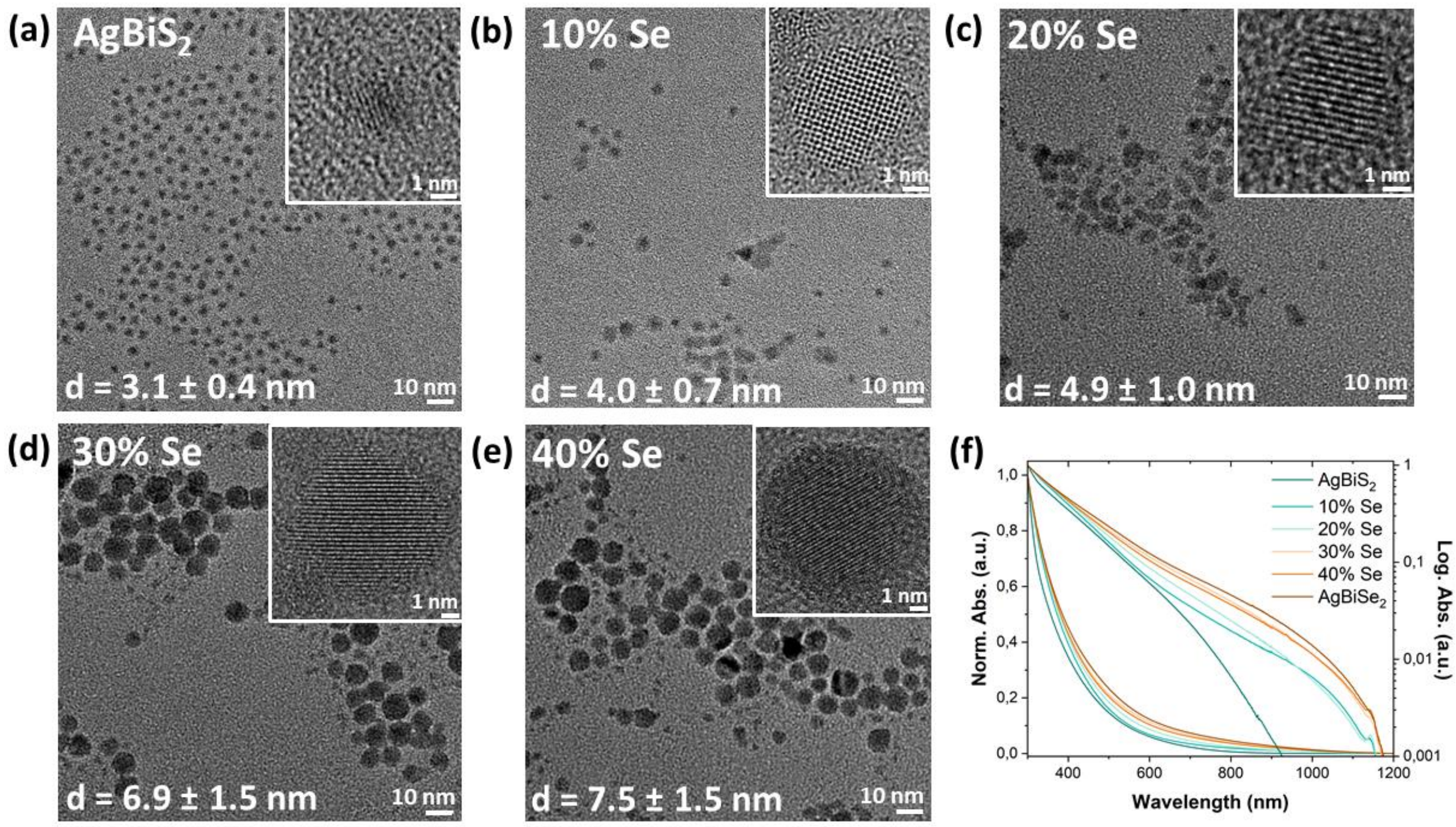

Figure S10. Characterization of $AgBiS_2$ and $AgBi(S_{1-x}Se_x)_2$ NCs, synthesized with La_2S and La_2S:La_2Se mixtures respectively, with varying ratios of S:Se. (a) TEM image of synthesized $AgBiS_2$ NCs. (b) TEM image and HRTEM inset of $AgBi(S_{1-x}Se_x)_2$ NCs with (b) 10%, (c) 20%, (d) 30%, and (e) 40% Se content. (f) Optical absorbance spectra of previously mentioned

synthesized NCs with varying Se contents, normalized and plotted in both linear and logarithmic scales.

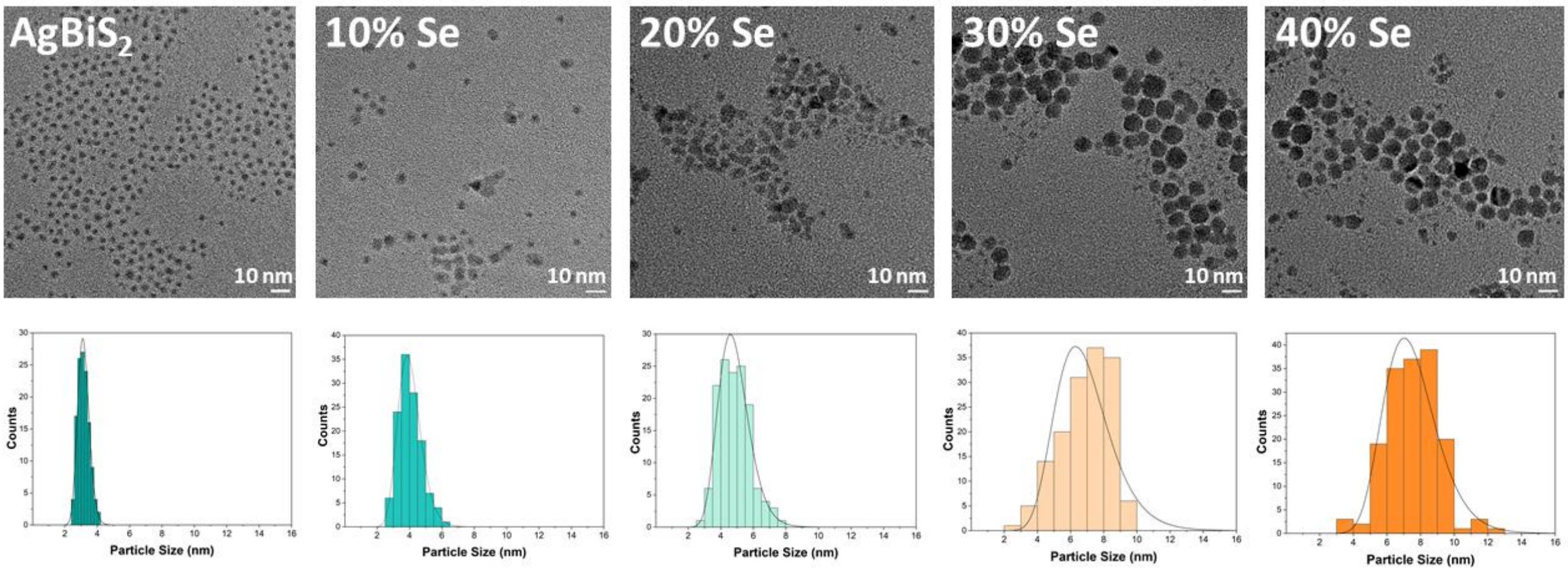

Figure S11. Particle size distributions of La_2S-based $AgBiS_2$ and $AgBi(S_{1-x}Se_x)_2$ NCs with different Se contents.

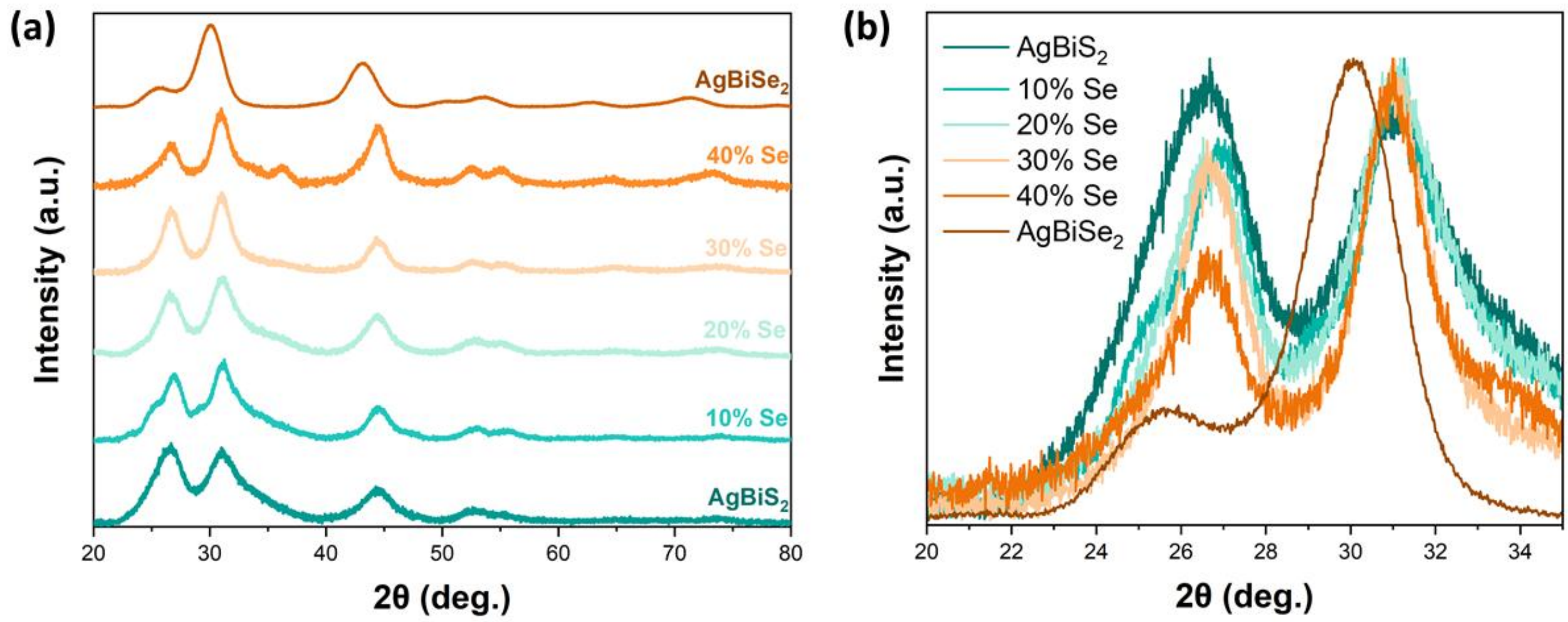

Figure S12. Structural analysis of La_2S-based NCs. (a) XRD patterns of synthesized $AgBi(S_{1-x}Se_x)_2$ NCs with varying contents of selenium. (b) Magnified XRD patterns, depicting the shifts of the two most prominent XRD peaks of synthesized $AgBi(S_{1-x}Se_x)_2$ NCs for varying contents of selenium.

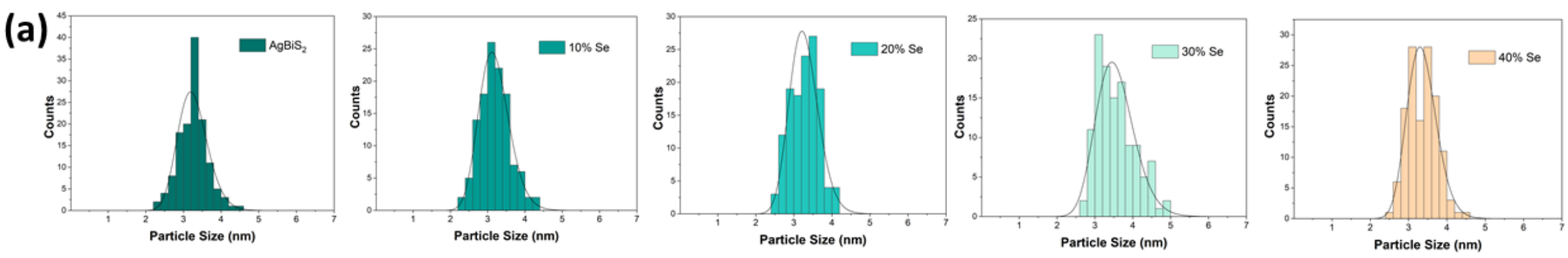

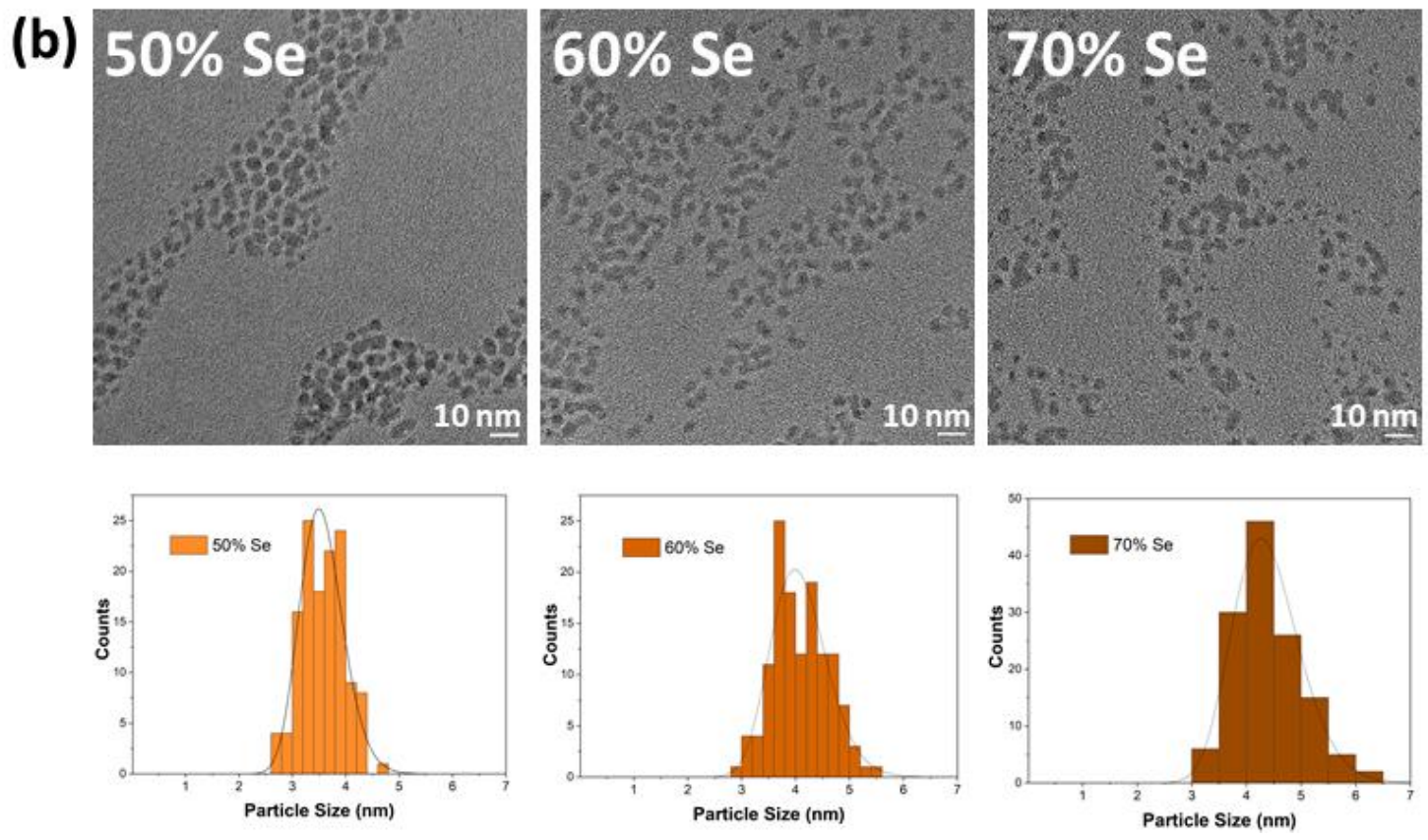

Figure S13. (a) Particle size distributions of $(TMS)_2S$-based $AgBiS_2$ and $AgBi(S_{1-x}Se_x)_2$ NCs with different Se contents, up to 40% selenium content. (b) TEM images and particle size distributions of NCs with 50-70% selenium content.

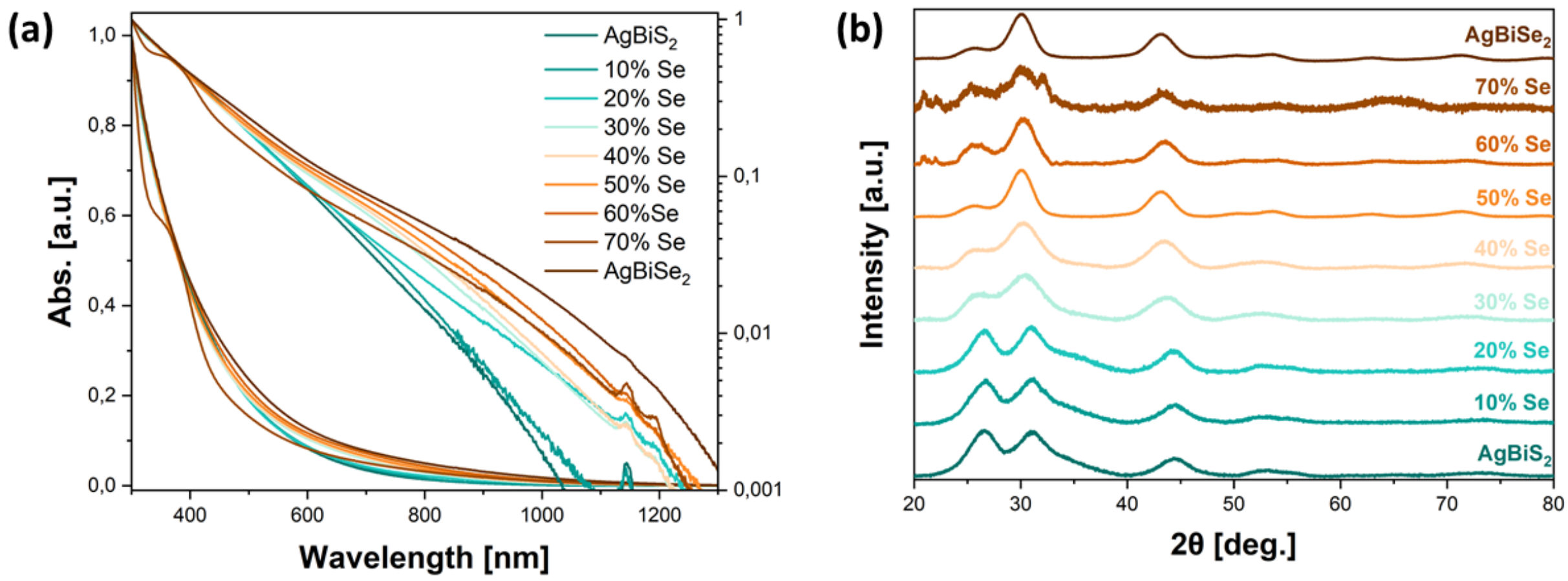

Figure S14. Characterization of $(TMS)_2S$-based $AgBi(S_{1-x}Se_x)_2$ NCs. (a) Optical absorbance spectra of NCs, up to 70% selenium content, plotted in linear and logarithmic scales. (b) XRD patterns of NCs, up to 70% selenium content.

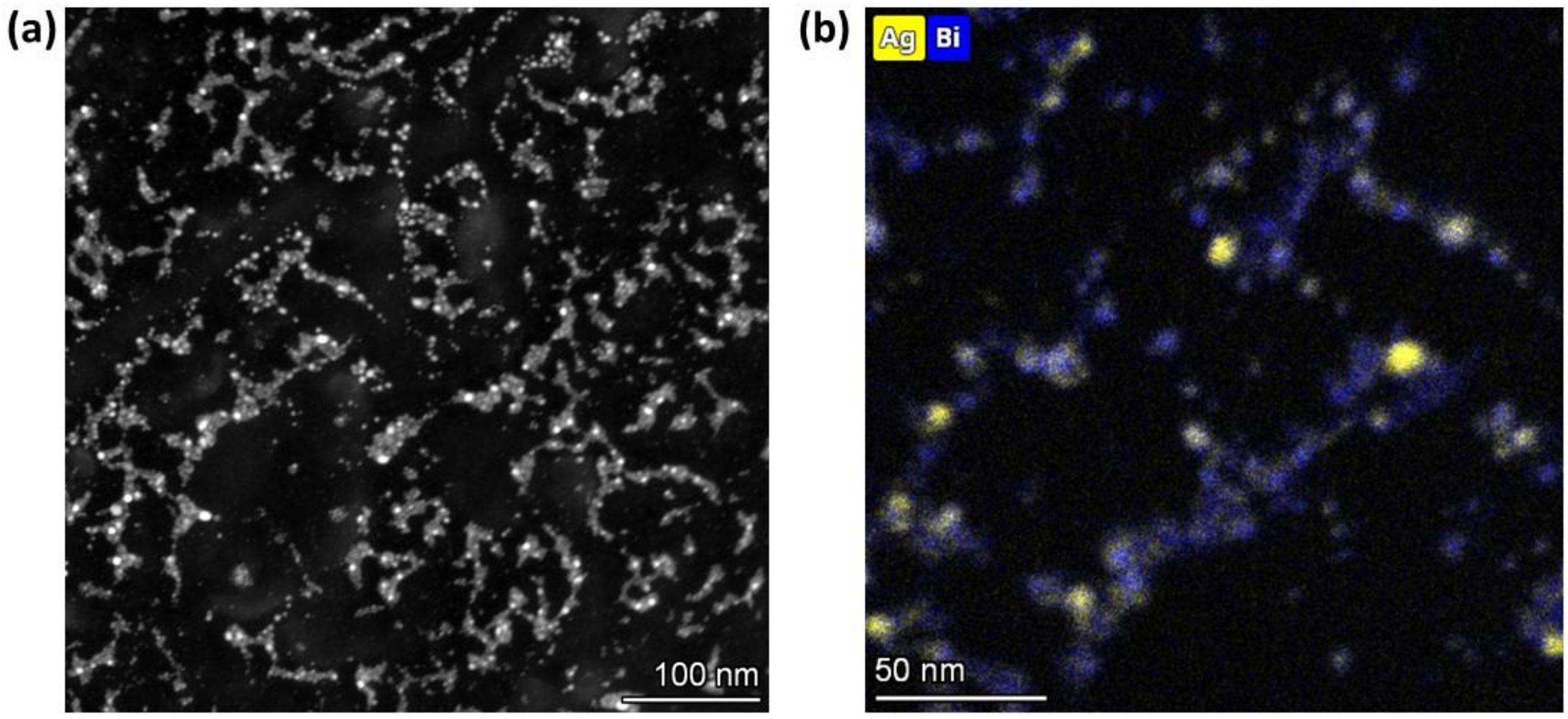

Figure S15. Characterization of $(TMS)_2S$-based $AgBi(S_{1-x}Se_x)_2$ NCs with 30% selenium content. (a) HAADF-STEM image of NCs. (b) Composite map of Ag and Bi.

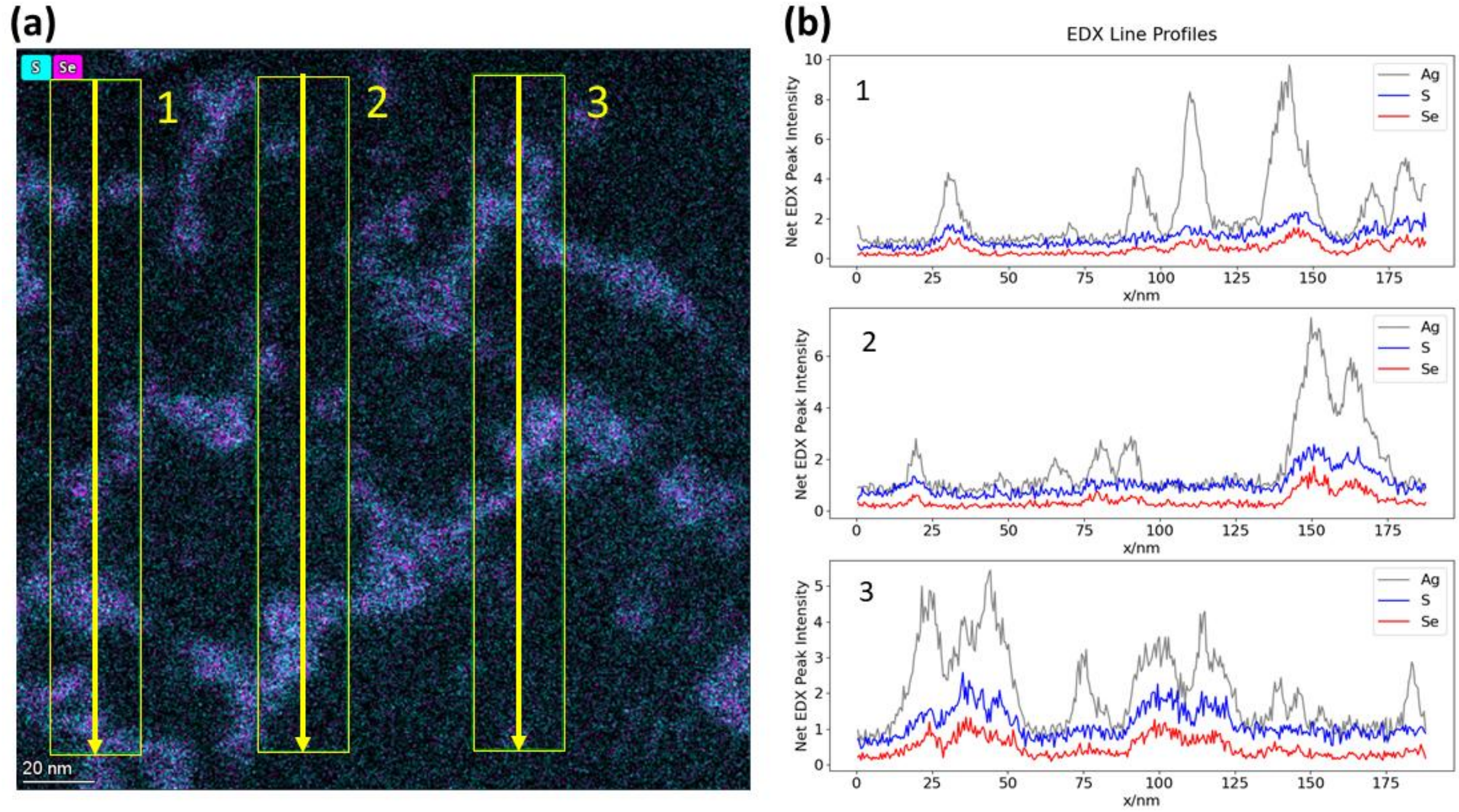

Figure S16. (a) EDX elemental map as composite images of Se (magenta) and S (cyan) background subtracted intensities. (b) Vertical line profiles as indicated in (a) showing the horizontally over 25nm averaged intensities for Se (red), S (blue) and Ag (gray).

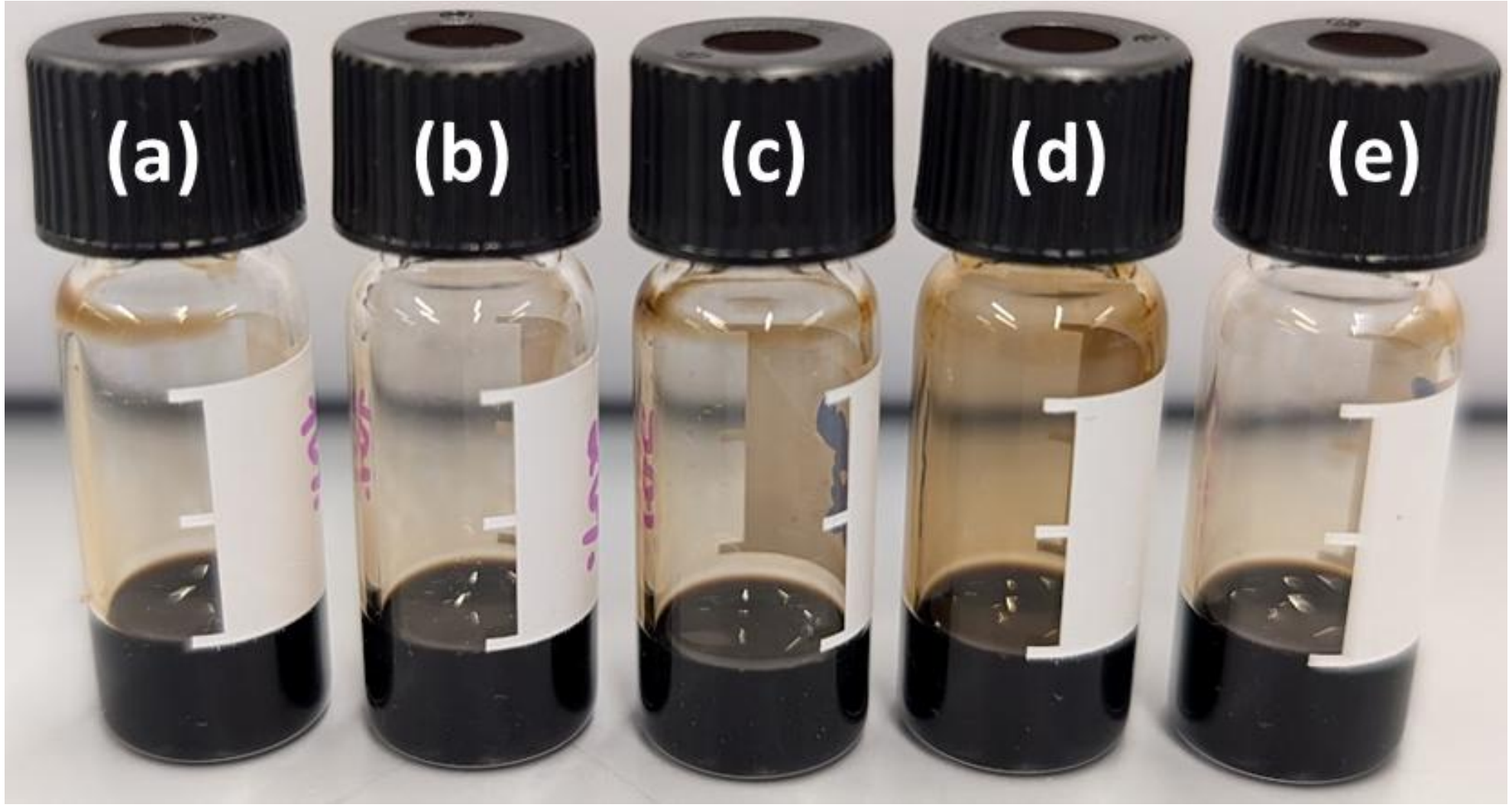

Figure S17. Synthesized $(TMS)_2S$-based $AgBi(S_{1-x}Se_x)_2$ NCs with different selenium contents, stabilized with 1-DDT. (a) 10% Se. (b) 20% Se. (c) 30% Se. (d) 40% Se. (e) 100% Se.

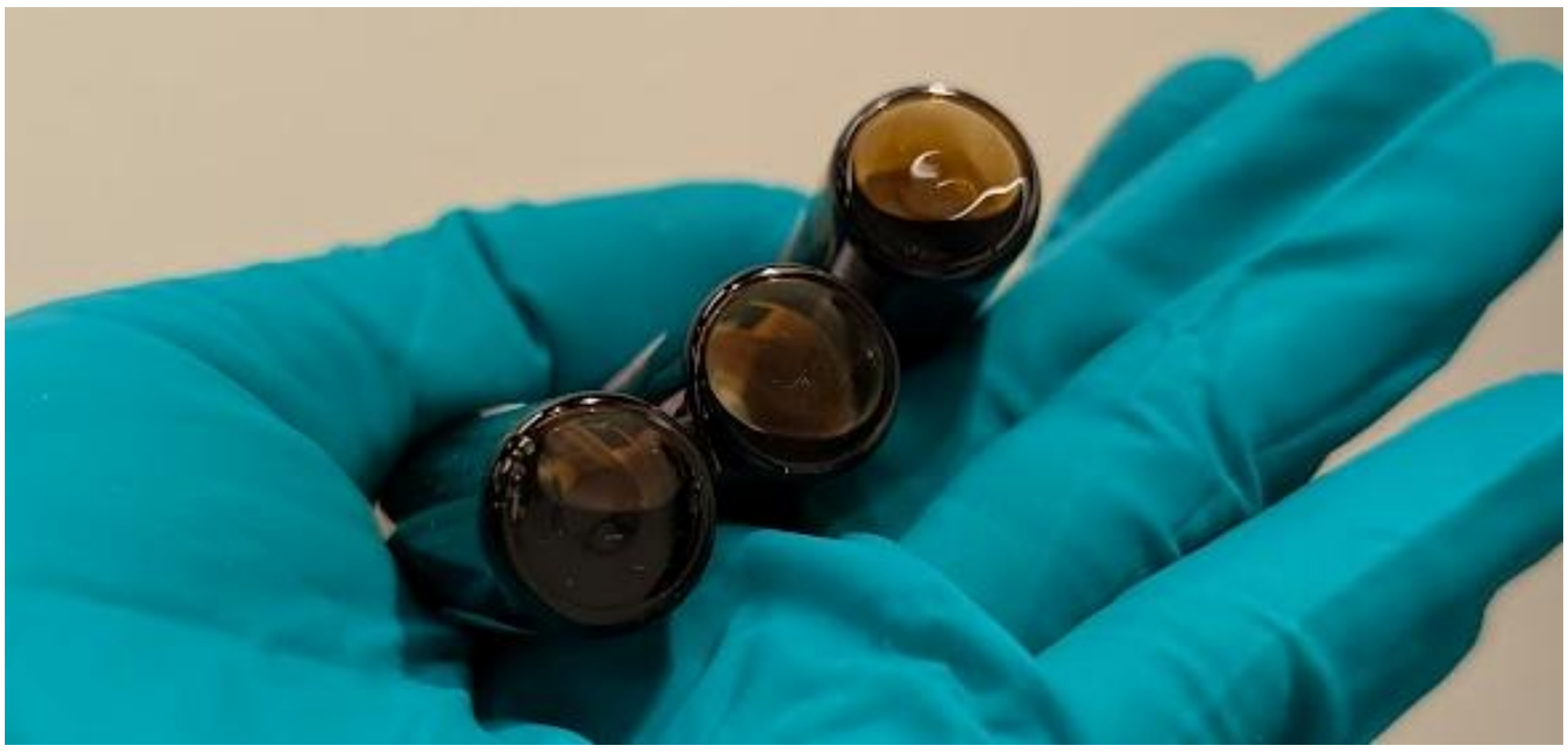

Figure S18. Synthesized $(TMS)_2S$-based $AgBi(S_{1-x}Se_x)_2$ NCs with different selenium contents, several weeks after synthesis.

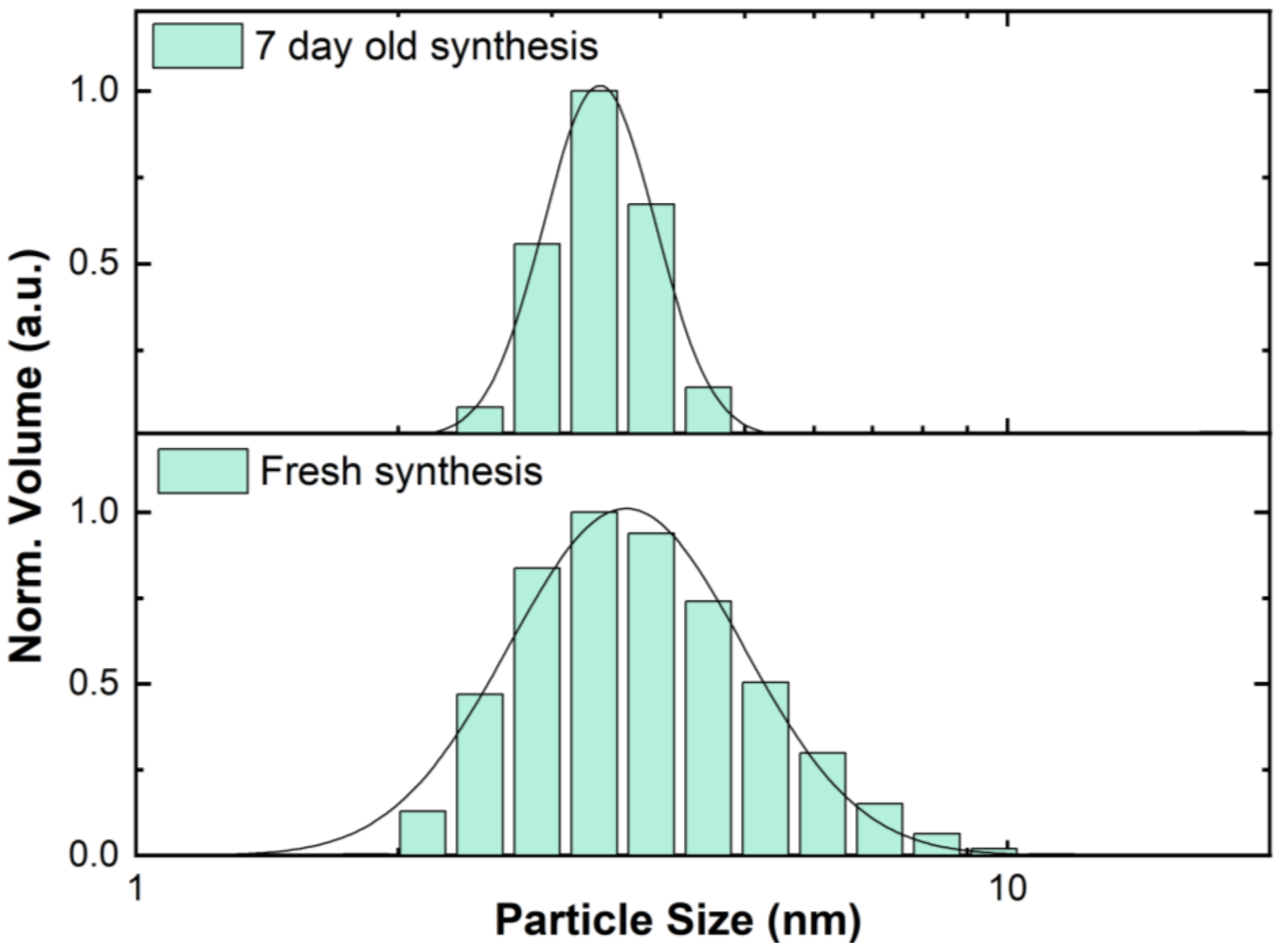

Figure S19. Dynamic light scattering (DLS) measurements of $(TMS)_2S$-based $AgBi(S_{1-x}Se_x)_2$ NCs with 30% selenium content, both freshly synthesized and aged for 7 days.

Table S1. ICP-OES analysis results of $(TMS)_2S$-based $AgBi(S_{1-x}Se_x)_2$ alloyed NCs, given in µg per 15 ml of solution.

Sample Name	Ag (µg)	Bi (µg)	Se (µg)	S (µg)
10% Se	29.47	35.94	1.88	17.86
20% Se	31.46	33.75	4.38	16.84
30% Se	22.68	26.95	6.11	11.16
40% Se	30.41	33.54	9.55	8.49

Table S2. ICP-OES analysis results of La_2S-based $AgBi(S_{1-x}Se_x)_2$ NCs, given in µg per 15 ml of solution.

Sample Name	Ag (µg)	Bi (µg)	Se (µg)	S (µg)
10% Se	29.92	18.61	2.03	6.34
20% Se	19.24	14.44	3.21	4.40
30% Se	19.69	18.77	4.01	4.77
40% Se	17.75	17.58	3.97	4.50
